# Construction of a physical fitness evaluation index system and model for high-level freestyle skiing aerials athletes in China

**DOI:** 10.1371/journal.pone.0295622

**Published:** 2023-12-08

**Authors:** Youwei Yao, Xuesong Niu

**Affiliations:** 1 School of Sports Training, Shenyang Sport University, Shenyang, China; 2 School of Social Sports, Shenyang Sport University, Shenyang, China; University of Study of Bari Aldo Moro, ITALY

## Abstract

**Objective:**

This study aims to enhance the competitive level of Chinese freestyle skiing aerials athletes by developing a specialized physical fitness evaluation index system and model tailored for high-level Chinese athletes. This system intends to provide theoretical references and training monitoring schemes in preparation for the 25th Milan Winter Olympics.

**Methods:**

A study was conducted on 29 high-level Chinese freestyle skiing aerials athletes. Physical fitness test indexes were selected using a literature review, expert interviews, and questionnaire surveys, and athletes were tested. Athletes were ensured to be in optimal physical condition before testing. Based on the test results, the representative indexes of the evaluation system are finally determined by combining R-type clustering analysis, multiple linear regression analysis. Determine index weights through weight questionnaires and normalization, and develop evaluation standards through methods such as percentile counting and weighted scoring.

**Results:**

Physical fitness evaluation system for Chinese freestyle skiing aerialists includes three aspects: evaluation index, index weight, and evaluation standard. The evaluation indexes include 3 first-level, 11 second-level, and 11 third-level indexes of body form, physiological function, and physical quality. In the evaluation weight, physical quality is ranked first, and physiological function and body form rank second and third, respectively. The evaluation standard consists of a scoring evaluation standard and a rating evaluation standard. Based on the index system, this study constructs the general and ideal physical fitness model of China’s high-level freestyle aerials athletes.

**Conclusion:**

The constructed physical fitness evaluation system effectively represents physical fitness development status of high-level freestyle skiing aerials athletes, providing a basis for creating personalized training plans. The established model serves as a reference for athletes’ physical fitness development objectives.

## Introduction

Freestyle skiing aerials is known as the “snow gymnastics project”; stability, difficulty, accuracy, and beauty are the remarkable characteristics of the sport. The sport plays a pivotal role in the Chinese delegation’s preparation for the Winter Olympics. China’s freestyle skiing aerials athletes at the 24th Beijing Winter Olympics had the best record of two gold and one silver medals, writing a new chapter for China’s ice and snow sports.

Freestyle skiing aerials competitions consist of acrobatic jumps, stressing takeoff, height, and distance (referred to as “air”), proper style, execution, and precision of movement (referred to as “form” and “landing”). The technical maneuvers of this sport include single, double, and triple movements. With the development of freestyle skiing aerial competitions worldwide, the competitive strength of the participants is rising. Triple movements have become the mainstay of the sport’s athletes competing for championships. Studies have shown that the success rate of aerial skill landing movements decreases with increasing technical difficulty [1]. The stability of the landing movement is an important link to improving the movement’s overall technical success rate. It is also the most basic and important measure of the difficult movement quality of freestyle skiing aerials athletes. A failed landing motion affects the final score and increases the probability of injury. Two studies on athletes’ injuries in two consecutive Winter Olympics in 2010 [2] and 2014 [3] show that the injury rate of freestyle skiing is high, and landing impact is an important factor leading to injury. The knee joint is the most vulnerable part of the body due to the high impact force of landing caused by the high takeoff height. Different landing states have different forces on the knee, and backward and forward landing increase the risk of injury to the anterior cruciate ligament (ACL) and posterior cruciate ligament (PCL), respectively [4]. Studies have shown that immediate vertical impact can damage the knee cartilage, while long-term recurrent shocks can cause strain on the stress concentration area of the cartilage [5]. The knee bends to an angle, and the instant valgus of the knee can lead to ligament damage [6–9]. Freestyle skiing aerial skills are assigned scoring sports with a significant risk coefficient. The sport requires athletes to complete a variety of complex horizontal axis rotation and vertical axis flipping movements and requires high executive control ability [10]. The difficulty coefficient of the athlete’s selected technical action determines the takeoff height. Triple movements require somersaults in the air above 10 meters. Any mistake in any step in the movement may cause an injury [11, 12].

According to the current problems of Chinese athletes in training or competition (unstable landing) and in combination with the competitive characteristics and development trend of aerial skills, physical fitness has become increasingly prominent in freestyle skiing aerialists [13, 14]. Intensive training to strengthen athletes’ core muscles can optimize somersault technique and landing stability [15]. Good physical fitness has become a necessary support and guarantee for athletes to improve their competitive ability and reduce sports injuries [16]. In particular, the improvement of strength quality plays a vital role in reducing the occurrence of injuries [17–20]. Improving lower limb muscle group strength can relieve the pressure on the knee joint and prevent sports injury [21–23].

To prevent injury and improve performance, athletes need to be evaluated to provide coaches and trainers with a basis for making decisions, which begins with an assessment of athletes’ physical fitness. Physical fitness refers to the essential ability of athletes and is a crucial component of competitive ability. To grasp the physical fitness of athletes at a point in time, coaches need to make a scientific evaluation of their physical fitness. According to the evaluation results, the coaches determine the strengths and weaknesses of the athletes’ physical fitness and then formulate a practical plan to make the training process more targeted. In addition, there is evidence that the evaluation of athletes’ physical fitness is a method to identify their sports injury risk factors [24].

In this study, we hypothesize that by implementing a series of specific physical fitness assessment indexes, we can predict the performance of Chinese high-level freestyle skiing aerials athletes. Therefore, this study will establish a physical fitness evaluation index system and model applicable to Chinese high-level freestyle skiing aerials athletes and explore the relationship between the system and athletes’ competitive performance. Through this study, we can provide a theoretical reference for the physical fitness training of Chinese freestyle skiing aerials athletes to enhance their competitive ability at the 25th Milan Winter Olympics.

## Materials and methods

### Participants

According to the physical fitness evaluation system established by the institute, the first-tier and second-tier athletes of freestyle skiing aerialists from the national training team for the 24th Beijing Winter Olympics were tested. A total of 29 athletes were enrolled, divided into the Elite Group (6 men, 5 women) and the Excellent Group (9 men, 9 women). The specific information on the athletes is shown in Table 1. The best results of the Elite Group’s male and female athletes were the top three finishes in the Winter Olympics, World Championship, or World Cup, and the best results of the Excellent Group’s male and female athletes were the top three finishes in competitions in China. All athletes participating in the testing were informed about all testing procedures, and written informed consent was obtained from all participants. The study received ethical approval from the Ethics Committee of Shenyang Sport University (No. 10095).

**Table 1:** Basic information of athletes in the freestyle skiing aerials national training team.

			Calendar Age (yrs)		Training age (yrs)		Height (cm)		Weight (kg)	
Group	Sample (n)		Male (M± SD)	Female (M± SD)	Male (M± SD)	Female (M± SD)	Male (M± SD)	Female (M± SD)	Male (M± SD)	Female (M± SD)
Elite Group	5	6	23.48±4.40	24.61±4.20	10.56±4.32	10.20±5.61	174.15±2.14	161.90±3.94	68.57±3.51	56.22±3.52
Excellent Group	9	9	19.56±2.29	20.22±3.67	7.85±3.61	8.34±4.59	175.53±4.99	158.78±3.46	69.21±4.82	55.37±5.92

### Study design

By consulting and collecting the literature related to the research direction of this paper, we systematically grasped the overview of the research on this topic and the latest research and development. The search included both written and web material. In this study, we not only prioritized the review of a series of professional literature pertaining to freestyle skiing aerials, especially those focusing on specialized physical quality [15, 16, 25–28] and monitoring of physical function indexes [29–32], but also referred to books related to sports physiology [33, 34], training science [35], and talent scouting [36, 37]. These materials provide a solid theoretical foundation for the research delineated in this paper.

To further understand the characteristics of freestyle skiing aerials and the basic characteristics of athletes’ physical fitness, we visited experts in freestyle skiing aerials at the Beijing Sports University and Shenyang Institute of Physical Education (n = 6). In addition, we also visited the national training team and provincial aerials coaches (n = 10), focusing on the selection of physical fitness test indexes, details of physical fitness test and evaluation methods, and other related issues and consulted these experts. This study is mainly in the form of interviews and telephone communications (The interview outline can be found in S1 Appendix). On the basis of a literature review and expert consultation, this article designed the “scoring scale of the physical fitness evaluation index of China’s high-level freestyle aerials athletes” and invited a total of 12 aerials coaches, referees and scientific researchers to participate in two rounds of surveys on the importance of the evaluation index. Of the 12 questionnaires distributed in each survey round, all 12 were returned. The index focused on body form, physiological function and physical quality for screening and identifying competitive athletes. According to the first round of responses to the questionnaire, the indexes modified and a second-round questionnaire was developed (see the S2 Appendix). Responses to this survey established the test indexes. Finally, statistical optimization established a more scientific physical fitness evaluation index. To determine the weight of each representative index in the constructed physical fitness evaluation system, this study compiled the “weight table of the physical fitness evaluation index of China’s high-level freestyle aerials athletes” and distributed it to relevant experts to assign a weight to each item of the physical fitness index. A total of 14 surveys were distributed and recovered (see the S3 Appendix).

To assess the validity of the questionnaires, this study enlisted 12 experts to evaluate the “Physical Fitness Evaluation Index Rating Scale” of whom 91.67% were either “satisfied” or “very satisfied”. Additionally, six experts with senior titles were selected to assess the “Physical Fitness Evaluation Index Weight Scale,” where 83.33% were “satisfied” and an additional 8.66% were “very satisfied” (see the S4 Appendix). This study used the “retest method” to test the reliability of the index weight scale questionnaire: (1) After the questionnaire was first distributed and filled in, six people were randomly selected from the list of experts who responded to the first survey and asked to retake the survey two weeks later; (2) The results of the two forms were analyzed with statistical software. The retest reliability coefficients of the first and second surveys were 0.87 and 0.89, respectively, indicating that the questionnaire had high reliability.

### Setting

In June 2020, we recruited athletes as participants for this study and simultaneously conducted data collection for physical fitness indexes, ensuring the optimal physical condition of these athletes prior to testing. The body form and physical quality parts of the testing were completed at the Qinhuangdao Training Base under the General Administration of Sport of China. The morphology and physical quality tests were carried out at the physical fitness training hall and track field at the Qinhuangdao Base by coaches and researchers. The physiological function tests were done at the Function Testing Center by the research experts with the team. Subsequently, we started writing the paper in April 2021 and completed the initial draft in December 2021. During this period, we conducted data analysis and, based on the results, established a physical fitness evaluation index system and model involving three aspects: body form, physiological function, and physical quality.

During the data collection process, we were able to access some personal identification information of the participants, including names, birth dates, etc. This information was necessary for data statistics in physical fitness indexes testing. However, we assure that this information was only used in the data statistics process and was not disclosed in any public papers or reports. To protect the privacy of the participants, all identifiable information was handled with strict confidentiality and security measures.

### Procedures and protocols

#### Morphological characteristics

To diagnose the body morphology characteristics of aerials athletes, the body morphology test method strictly follows the test rules of Sports Measurement and Evaluation [37]. It is completed by special personnel with standard instruments. Body composition was measured using a multifrequency segmented bioelectrical impedance body composition tester (model: InBody270, Yinbadi Co., Ltd., CN).

#### Physiological characteristics

To measure the physiological function characteristics of aerials athletes, one anaerobic power bicycle (model MONARK837, Switzerland), one exercise cardiopulmonary function test system (model MAXII, USA) and one bicycle ergometer (model: MONARK839E, Switzerland) were used in this study. The testing process of physiological and biochemical indexes was completed by special personnel using standard instruments: one hemoglobin analyzer (model: XF-IB, CN), one blood urea semiautomatic biochemical analyzer (model: BT-1904C, CN), one red blood cell analyzer (model: BECKMAN STKS, CN), one cortisol analyzer (model: DSL-10-67100, USA), and one serum testosterone analyzer (model: DSL-10-4000, USA).

#### Athletic quality characteristics

To test the physical quality characteristics of aerial skills athletes, barbell tablets (Zhang Kong barbell Manufacturing Co., Ltd., CN), barbell bars (Zhang Kong barbell Manufacturing Co., Ltd., CN), hard medicine balls (Jia You sports and leisure products Co., Ltd., CN), logo discs (Jia You sports and leisure products Co., Ltd., CN), balance pads (Jia You sports and leisure products Co., Ltd., CN), yoga mats (Jia You sports and leisure products Co., Ltd., CN), tapes (Great Wall seiko Industrial Co., Ltd., CN) and ribbed wooden frames (WaterRower Co., Ltd., Germany) were used in this study. For timing purposes, electronic stopwatches (SEIKO, SVAJ007, Japan) and an optical door timing system (SmartSpeed, Fusion Sport Ltd., Australia) were used. The test methods were clearly presented in **S5 Appendix**.

### Data analyses

The raw data were stored in Microsoft Excel format, and the database was established. Mathematical statistics were processed by SPSS22.0 software. Some conventional statistical methods were used in data analysis, such as Delphi calculation, weight coefficient calculation, index value model calculation, cluster analysis method, multiple linear regression analysis, and percentile method.

## Results

### Construction of the physical fitness evaluation index system for aerials athletes

#### Determination of the physical fitness evaluation indexes of aerials athletes

This study started with the three structural elements commonly recognized in the concept of physical fitness [35, 38]: 3 first-level indexes, 13 second-level indexes, and 36 third-level indexes of body form, physiological function, and physical quality, which were determined through the literature review, expert interviews, and logical analysis. This formed the initial comprehensive evaluation system of special physical fitness indexes for China’s high-level freestyle aerials athletes.

After the preliminary screening, the Delphi method was used to further evaluate the indexes. The specific steps were as follows [39–41]: (1) Designed the expert questionnaire with the preliminarily selected indexes, and marked the importance and corresponding scores for each index so that the experts could score each index in an easy way; (2) Invited experts and coaches to screen the index and assign values to the items; (3) Calculated the average value by mathematical statistics based on the scores; (4) Selected the indexes with mean values ≥ 4 and coefficient of variation < 0.25 as further statistical screening indexes and as the test items of physical fitness indexes for this study (Table 2).

**Table 2:** Test item model of the physical fitness index of freestyle skiing aerials athletes.

Primary indexes	Secondary indexes	Tertiary indexes
Body form (15 items)	Length	Achilles tendon length, Upper limb length, Lower limb length, Height (4 items)
	Width	Shoulder width, Pelvis width (2 items)
	Girth	Thigh circumference, Calf circumference, Waist circumference (3 items)
	Body composition	Body fat percentage, Quetelet index, Waist-hip ratio, Body mass index, Body weight, Fat-free body weight (6 items)
Physiological function (9 items)	Cardiopulmonary function	Maximal oxygen intake, Relative maximum oxygen uptake (2 items)
	Anaerobic capacity	Maximum anaerobic power, Relative maximum anaerobic power (2 items)
	Oxygen usage ability	Hemoglobin, Red-cell count (2 items)
	Energy metabolism and endocrine system	Blood urea, Serum testosterone, Serum cortisol (3 items)
Physical quality (14 items)	Strength	Squat up, Pull up, Power clean, Standing long jump, Side throw (left and right), Back throw, Single-leg triple jump (left and right), Squat on the balance pad with the barbell raising, Quick v-up (11 items)
	Speed	30-meter sprint (1 item)
	Endurance	12-minute run (1 item)
	Agility	Agile running (1 item)

The “Expert Authority Level” can reflect the reliability of the questionnaire. The degree of authority of the experts can be assessed through the authority coefficient, which is calculated as the arithmetic mean of the judgment basis and the degree of familiarity with the selected index (Authority Coefficient = (Judgment Basis + Degree of Familiarity) / 2). A higher value of the authority coefficient signifies a higher level of expert authority. Typically, an authority coefficient value of ≥ 0.70 is regarded as indicating a high degree of authority[42]. The survey data from this study revealed that the authority coefficients for the first and second rounds of the questionnaire were 0.86 and 0.89, respectively, indicating that the selected experts in this research possess a high degree of authority (see the S2 Appendix).

After the evaluation indexes were selected by experts, we conducted physical fitness tests on 29 high-level athletes specializing in freestyle skiing aerials and obtained specific data on various indexes. An R-type cluster analysis was performed on the data, categorizing it based on body form, physiological function, and physical quality [43, 44] (see the S6 Appendix). Upon completing the cluster analysis, a representative index was selected from each category as the final evaluation index. If only one index remained after classification, it became the representative index of that category. If two indexes remained, one was selected by integrating specialized characteristics and relevant professional knowledge. For categories with three or more indexes, a multivariate linear regression analysis was conducted. An index within a category served as the dependent variable, while the remaining indexes functioned as independent variables. A forced entry analysis method was used to determine the coefficient of determination of the dependent variable. This procedure was repeated to calculate the coefficient of determination for each index within a category, ultimately selecting the index with the highest coefficient as the representative index [44] (see the S6 Appendix).

After obtaining specific data for 15 body form indexes through testing, we conducted a cluster analysis using SPSS 22.0 statistical software. Through cluster analysis and in combination with relevant theoretical knowledge [37], we categorized the 15 body form indexes into four groups: body length, body width, body circumference, and body composition, as illustrated in Table 3. Subsequently, we selected a typical representative index within each classification. The classification indexes for body length, body circumference, and body composition all consisted of three or more indexes. Therefore, when selecting representative indexes, we conducted regression analyses respectively and chose the indexes with the highest coefficients of determination as the representative indexes. In the category of body width, shoulder width and pelvic width were the two selected indexes. Given the characteristics of the freestyle skiing aerials event, athletes require a higher emphasis on pelvic width compared to shoulder width. A narrower pelvis facilitates the completion of technical moves [45]. When athletes initiate a spin after takeoff, a relatively narrower pelvis reduces the radius of pelvic rotation, resulting in a smaller moment of inertia around the longitudinal axis, which facilitates achieving the maximum rotational frequency. This aids freestyle skiing aerials athletes in maintaining a faster rotational speed during spins, setting a prerequisite for attempting new technical moves [46, 47]. Consequently, this study chose pelvic width as the representative index for body width. Ultimately, after filtering through the 15 physical morphology indexes, we established an evaluation system for the Body form indexes of high-level male and female freestyle skiing aerials athletes, which includes four items: Achilles tendon length, pelvic width, waist circumference, and the Quetelet index.

**Table 3:** Cluster analysis and naming results of body form indexes in freestyle skiing aerials athletes.

Gender (n)	Naming by Clustering	Corresponding indexes
Male 15) /Female (14)	Body length	Achilles tendon length, Upper limb length, Lower limb length, Height
	Body width	Pelvis width, Shoulder width
	Body circumference	Waist circumference, Calf circumference, Thigh circumference
	Body composition	BMI, Waist-hip ratio, Quetelet index, Body fat percentage, Fat-free body weight, Body weight

After acquiring specific data on 9 physiological function indexes through testing, we utilized the SPSS 22.0 statistical software for cluster analysis. Following the cluster analysis and incorporating pertinent knowledge [29], we divided the 9 physiological function indexes into three categories: anaerobic capacity, cardiopulmonary performance, and exercise biochemical ability (as depicted in Table 4). Thereafter, we selected a typical representative index within each category. In the anaerobic capacity category, there were two indexes. Maximum anaerobic power is an absolute value index, directly reflecting the athlete’s maximum power output capability, while relative maximum anaerobic power is a relative value index, illustrating the relationship between the athlete’s power output ability and their weight by dividing the maximum anaerobic power by weight. The cardiopulmonary performance category also contains two similar indexes: maximum oxygen uptake (absolute value) and relative maximum oxygen uptake (relative value). For competitive sports disciplines where weight control is essential, like freestyle skiing aerials [32], relative indexes hold more significance than absolute indexes [48, 49]. Consequently, this study chose relative maximum anaerobic power as the representative index for anaerobic capacity, and relative maximum oxygen uptake as the representative index for cardiopulmonary performance. The exercise biochemical ability category encompasses more than three indexes. Therefore, when selecting the representative indexes, we conducted regression analysis and chose the indexes with the highest coefficients of determination as the representative indexes. After filtering through the 9 physiological function indexes, we ultimately established an evaluation system for the physiological function indexes of high-level male and female freestyle skiing aerialists, encompassing three items: relative maximum anaerobic power, relative maximum oxygen uptake, and hemoglobin.

**Table 4:** Cluster analysis and naming results of physiological function indexes in freestyle skiing aerials athletes.

Gender (n)	Naming by Clustering	Corresponding indexes
Male (15) /Female (14)	Aerobic ability	Relative maximum anaerobic power, Maximum anaerobic power
	Cardiopulmonary performance	Relative maximum oxygen uptake, Maximal oxygen intake
	Exercise biochemical ability	Serum cortisol, Serum testosterone, Blood urea, Red-cell count, Hemoglobin

After obtaining specific data on 14 physical quality indexes through testing, we employed the SPSS 22.0 statistical software for cluster analysis. Through the cluster analysis and integrating relevant knowledge [35], we categorized the 14 physical quality indexes into four categories: limb strength, core strength, speed-agility, and aerobic capacity (as shown in Table 5). Subsequently, we selected a typical representative index within each category. Both the limb strength and core strength categories contained more than three indexes. Hence, when choosing the representative indexes in these categories, we conducted regression analyses separately and selected the indexes with the highest coefficients of determination as the representative indexes. In the speed-agility category, there were two indexes: agile running and 30-meter sprint. Considering the uniqueness of the freestyle skiing aerials discipline, the 30-meter sprint seems particularly appropriate. This metric not only accurately reflects the agility capabilities of an athlete’s limbs but also unveils the energy metabolism characteristics during critical technical phases (somersault in the air and landing stages) of the discipline. In this pivotal phase, the time of force generation is generally 2–3 seconds, mainly relying on the phosphagen system for energy provision. Therefore, selecting the 30-meter sprint run as the representative index is more aligned with the actual demand characteristics of this sport discipline [16]. Ultimately, after statistical screening of the 14 physical quality indexes, this study established an evaluation system for the physical quality of high-level male and female freestyle skiing aerials athletes, comprising four key indexes: power clean, squat on the balance pad with the barbell raising, 30-meter sprint, and 12-minute run.

**Table 5:** Cluster analysis and naming results of physical quality indexes in freestyle skiing aerials athletes.

Gender (n)	Naming by Clustering	Corresponding indexes
Male (15) /Female (14)	Limb strength	Squat up, Pull up, Power clean, Standing long jump, Single-leg triple jump (left), Single-leg triple jump (right)
	Core strength	Side throw(right), Side throw(left), Back throw, Squat on the balance pad with the barbell raising, Quick v-up
	Speed-agility	30-meter sprint, Agile running
	Aerobic capacity	12-minute run

#### Determination of the weight coefficient of the physical fitness evaluation index of aerials athletes

Weights were assigned to give relative importance to the factors being compared in the overall evaluation. This study invited 14 experts in the field of freestyle skiing aerials to assign weights to physical fitness indexes based on their professional knowledge and experience. The specific process is as follows: (1) Creation of an expert consultation form: Listing each index that requires weighting, and inviting experts to score each index based on their experience and knowledge, using a five-level scoring method (scores ranging from 1 to 5). (2) Collection and processing of questionnaires: Counting the number of identical scores for each index, and calculating the score of each index. The score calculation method involves multiplying each level of score by its frequency, and then summing the results. (3) Weight calculation: Using the summation and normalization method, the weights of each index were calculated (the results of the weight assignment are shown in Table 6).

**Table 6:** Special physical fitness evaluation index system and weight of high-level freestyle skiing aerials athletes in China.

Primary indexes	Weight (Male/Female)	Tertiary indexes	Weight (Male/Female)
A1 Body form	0.25/0.27	A1 Achilles tendon length	0.24/0.23
		A2 Pelvis width	0.23/0.22
		A3 Waist circumference	0.31/0.30
		A4 Quetelet index	0.22/0.25
B2 Physiological function	0.33/0.32	B1 Relative maximum anaerobic power	0.35/0.36
		B2 Relative maximum oxygen uptake	0.31/0.29
		B3 Hemoglobin	0.34/0.35
C3 Physical quality	0.42/0.41	C1 Power clean	0.30/0.27
		C2 Squat on the balance pad with the barbell raising	0.29/0.28
		C3 30-meter sprint	0.20/0.20
		C4 12-minute run	0.21/0.25

To verify the rationality of the weights, we created an “Expert Evaluation Form for the Weight Coefficients of Physical Fitness Evaluation Indexes” (S3 Appendix), and randomly selected 6 experts for a survey. 83.33% of experts approved the weight coefficients determined by the research, indicating that the obtained weights are relatively objective and accurate.

It can be seen from the weight of the first-level indexes in Table 6 that the physical quality of men and women in freestyle skiing aerials in China is high, with weight scores of 0.42 and 0.41, respectively. Their weight scores were higher than their physiological function and body form scores. This shows that athletic quality plays a key role in the success of aerials athletes and is dominant in developing their physical fitness level.

The circumference index of the four individual indexes (three-level indexes) had the highest value among the body form indexes. The weighted score was 0.30, indicating that the freestyle skiing aerials program placed a high demand on the core strength of athletes, which suggests that coaches should pay attention to the training of the waist and abdominal area in the training process. In the physiological function indexes, the relative maximum anaerobic power and hemoglobin were weighted more heavily, and these two indexes represent the athlete’s anaerobic capacity and the body’s ability to transport oxygen. This should be a reminder to coaches to improve these two aspects of ability in daily training. In the physical quality indexes, the indexes reflecting the lower limb explosive force, centrifugal buffer ability, and core stability ability in the unsteady state were weighted heavily, which indicates that the dominant physical qualities of freestyle skiing aerials athletes are lower limb strength and core strength. Therefore, the training of lower limbs and core aspects should be emphasized in physical training.

#### Establishment of physical fitness evaluation standards for aerials athletes

To master the difference in the special physical fitness of athletes of different levels in China, it is necessary to treat the difference in athletes’ physical fitness objectively and quantitatively. Therefore, it is essential to develop a relatively unified physical fitness evaluation standard for freestyle skiing aerials athletes. This process can quantify the physical fitness level of aerials athletes, provide a reference for subsequent training plan formulation, and make the plan more targeted and personalized.

Next, we will explore the process of formulating the scoring and rating standards for individual indexes of physical fitness. In this study, the percentile method [50, 51] was first adopted, and the 5-point scoring method was used to score each selected individual index to obtain the scoring interval and scoring standard of the individual index. The specific steps were as follows: (1) To determine the theoretical percentage of the number of people at each level, the number of people with intermediate grades should account for a relatively high percentage, while the number of people with high and low grades should account for a relatively low percentage (Table 7); (2) To determine the dividing points (P10, P25, P50, P75, P90) corresponding to each evaluation level, first, the minimum and maximum values of individual indexes were determined and the scores of the 10th, 25th, 50th, 75th, and 90th percentiles were calculated. Second, different grades were established (above P90, P75~P90, P25~P75, P10~P25, and below P10) and defined according to corresponding scores. More than 90% was considered excellent and given a score of 5 points; >75%~90% was defined as good, and the score was 4 points; 25%~75% was defined as a medium, and the score was 3 points; 10%~<25% was defined as low, and the score was 2 points; below 10% was defined as poor, and the score was 1 point. (3) Third, the scoring and rating criteria for individual indexes of special physical fitness of athletes in high-level freestyle skiing aerials were formulated, as shown in Table 8. Please note that the smaller numerical value of squat on the balance pad with the barbell raising and 30-meter sprint indicated a low optimal index, while the greater numerical value on other indexes indicated a high optimal index.

**Table 7:** Scoring and rating standards of special physical fitness indexes by percentile method.

Theoretical score (%)	Evaluation standard		Score	Rank
	High optimal index	Low optimal index		
10	Over 90%	Under 10%	5	Excellent
15	>75%~90%	10%~<25%	4	Good
50	25%~75%	25%~75%	3	Average
15	10%~<25%	>75%~90%	2	Poor
10	Under 10%	Over90%	1	Fail

**Table 8:** Scoring standards and grading standards of the single physical fitness index of China’s high-level freestyle skiing aerials athletes (male / female).

Evaluation Index	1 point (fail)		2 points (poor)	3 points (average)	4 points (good)	5 points (excellent)
	High optimal	Under 10%	10%~<25%	25%~75%	>75%~90%	Over 90%
	Low optimal	Over 90%	>75%~90%	25%~75%	10%~<25%	Under 10%
Achilles tendon length(cm)	<20.81/ <19.64		20.81~<21.41/ 19.64~<19.94	21.41–22.44/ 19.94–20.95	>22.44~22.83/ >20.95~21.42	>22.83/ >21.42
Pelvis width(cm)	<25.48/ <25.43		25.48~<25.90/ 25.43~<25.52	25.90~27.31/ 25.52~26.68	>27.31~28.76/ >26.68~27.16	>28.76/ >27.16
Waist circumference(cm)	<68.78/ <66.29		68.78~<70.99/ 66.29~<66.60	70.99–73.28/ 66.60–69.45	>73.28~73.66/ >69.45~72.71	>73.66/ >72.71
Quetelet index	<374.28/ <322.13		374.28~<376.33/ 322.13~<331.98	376.33~406.86/ 331.98~352.42	>406.86~424.72/ >352.42~373.86	>424.72/ >373.86
Relative maximum anaerobic power (w/kg)	<13.53/ <13.07		13.53~<14.02/ 13.07~<13.41	14.02–15.94/ 13.41–14.34	>15.94~16.78/ >14.34~14.86	>16.78/ >14.86
Relative maximum oxygen uptake (ml/kg/min)	<38.74/ <41.95		38.74~<40.72/ 41.95~<45.37	40.72-48/ 45.37–49.78	>48~50.20/ >49.78~51.06	>50.20/ >51.06
Hemoglobin(g/L)	<129/ <117		129~<140/ 117~<121	140-160/ 121–137	>160~165/ >137~142	>165/ >142
Power clean(kg)	<75/ <29		75~<80/ 29~<33	80-98/ 33–52	>98~103/ >52~64	>103/ >64
Squat on the balance pad with the barbell raising(s)	>14.09/ >20.49		14.09~>11.50/ 20.49~>17.96	11.50–9.61/ 17.96–11.20	<9.61~9.09/ <11.20~10.04	<9.09/ <10.04
30-meter sprint(s)	>5.12/ >5.50		5.12~>4.58/ 5.50~>5.15	4.58–4.02/ 5.15–4.72	<4.02~3.84/ <4.72~4.63	<3.84/ <4.63
12-minute run(m)	<2346/ <2062		2346~<2475/ 2062~<2213	2475~2805/ 2213~2620	>2805~2939/ >2620~2771	>2939/ >2771

Subsequently, we will delve into the process of formulating scoring and rating standards for the first-level index and comprehensive index of physical fitness. According to the above scoring standards, we can understand the scores of athletes in each index to grasp the position of each individual index and then realize the horizontal comparison of different athletes’ specific physical fitness levels. To comprehensively evaluate the physical fitness of athletes, it is necessary to evaluate the effect of each index on the specific physical fitness (i.e., the weight) on the basis of individual score evaluation. The formula for calculating the weighted score is as follows:

*N* = ∑*niwi* (ni represents the score of each index, and wi represents the weight of each index)

The following four methods should be adopted for the comprehensive scoring standard of the athletes’ special physical fitness in this sport: (1) The weighted score of every single index is calculated according to the different weights of every single index (three-level index) under each first-level index. (2) According to the weighted score of every single index, the score of each first-level index (unweighted) is calculated. (3) The weighted score of each first-level index is calculated according to the different weights of each first-level index. (4) Add and sum the weighted scores of the three first-level indexes to obtain the comprehensive physical fitness score. According to the above steps, the grade evaluation criteria of the individual index, the first index and the comprehensive index of the physical fitness of China’s high-level freestyle aerial skiers are determined (Table 9).

**Table 9:** Primary indexes of physical fitness, comprehensive index score and rating criteria of Chinese high-level freestyle skiing aerials athletes (male/female).

Evaluation Index		1 point (fail)	2 points (poor)	3 points (average)	4 points (good)	5 points (excellent)
		Under 10%	10%~<25%	25%~75%	>75%~90%	Over 90%
Primary indexes	Body form	<0.63/ <0.62	0.63~<0.69/ 0.62~<0.67	0.69~0.80/ 0.67~0.83	>0.80–0.85/ >0.83–0.91	>0.85/ >0.91
	Physiological function	<0.85/ <0.67	0.85~<1.03/ 0.67~<0.79	1.03~1.25/ 0.79~1.13	>1.25–1.31/ >1.13–1.26	>1.31/ >1.26
	Physical quality	<1.08/ <0.91	1.08~<1.13/ 0.91~<1.03	1.13~1.32/ 1.03~1.30	>1.32–1.56/ >1.30–1.51	>1.56/ >1.51
Comprehensive index		<2.80/ <2.39	2.80~<2.85/ 2.39~<2.58	2.85~3.32/ 2.58~3.26	3.32>3.53/ >3.26–3.46	>3.53/ >3.46

### Evaluation results of the physical fitness level of aerials athletes

The objective of this paper is to evaluate the physical fitness level of China’s high-level aerials athletes effectively and scientifically. Practice is the only criterion to test truth. The evaluation of high-level athletes can help us understand the current physical status of athletes and test whether the system built in this paper is reasonable and effective. By checking the rationality of the evaluation system, it can provide a reliable reference for the application and promotion of the evaluation system and evaluation criteria in the future. Therefore, after the construction of the index evaluation system, this paper evaluates the physical fitness level of the best male and female athletes in active service in China according to the scoring and rating standards established above.

#### Scoring results of physical fitness level of aerials athletes

First, the original score of each evaluation index of physical fitness was determined according to the test results of each index and the scoring criteria established previously. Then, the obtained score was multiplied by the weight of each index to obtain the weighted score of each index. Finally, the weighted score and total score of each athlete’s three individual performance elements were calculated (Tables 10, 11).

**Table 10:** Scores of primary physical fitness indexes and comprehensive index for first-tier freestyle skiing aerials athletes (weighted).

Males (n = 6)					Females (n = 5)				
Name	Body form	Physiological function	Physical quality	Comprehensive index	Name	Body form	Physiological function	Physical quality	Comprehensive index
W**	0.82	1.09	1.68	3.59	X**	0.94	1.28	1.85	3.94
Q**	0.79	1.31	2.01	4.11	X**	0.84	1.07	1.54	3.45
W**	0.64	1.31	1.39	3.34	X**	0.82	1.21	1.44	3.46
S**	0.83	1.09	1.38	3.30	K**	0.74	1.51	1.03	3.28
Y**	0.87	1.31	1.26	3.44	S**	0.81	0.98	1.32	3.11
J**	0.71	0.99	1.12	2.82					

**Table 11:** Scores of primary physical fitness indexes and comprehensive index for second-tier freestyle skiing aerials athletes (weighted).

Males (n = 9)					Females (n = 9)				
Name	Body form	Physiological function	Physical quality	Comprehensive index	Name	Body form	Physiological function	Physical quality	Comprehensive index
S**	0.69	1.09	1.09	2.88	L**	0.93	1.15	1.12	3.19
L**	0.70	1.19	1.26	3.15	L**	0.59	0.84	0.91	2.34
Y**	0.63	1.19	1.26	3.09	J**	0.73	0.64	1.12	2.49
L**	0.75	0.98	0.79	2.52	Y**	0.63	0.84	1.23	2.70
F**	0.60	1.08	1.13	2.81	D**	0.87	0.96	1.21	3.05
S**	0.92	0.76	1.26	2.94	S**	0.81	0.73	1.04	2.58
L**	0.70	1.42	1.18	3.29	Z**	1.06	0.62	0.91	2.59
G**	0.78	0.76	1.26	2.80	Y**	0.65	1.07	1.23	2.96
L**	0.75	1.09	1.07	2.91	L**	0.62	0.77	0.86	2.26

#### Rating results of physical fitness levels among freestyle skiing aerials athletes

According to the weighted score and total score of each physical ability element of athletes shown in Tables 7 and 8 and combined with the rating criteria developed above, the grade evaluation results of each athlete’s three physical ability elements can be obtained (Tables 12 and 13).

**Table 12:** Rating evaluation results of the physical fitness level of first-tier freestyle skiing aerials athletes.

Males (n = 6)					Females (n = 5)				
Name	Body form	Physiological function	Physical quality	Comprehensive index	Name	Body form	Physiological function	Physical quality	Comprehensive index
W**	Good	Average	Excellent	Excellent	X**	Excellent	Excellent	Excellent	Excellent
Q**	Excellent	Excellent	Excellent	Excellent	X**	Good	Average	Good	Good
W**	Poor	Good	Good	Good	X**	Average	Good	Good	Good
S**	Good	Average	Good	Good	K**	Average	Good	Average	Good
Y**	Excellent	Good	Good	Good	S**	Good	Average	Good	Average
J**	Good	Poor	Poor	Poor					

**Table 13:** Rating evaluation results of the physical fitness level of second-tier freestyle skiing aerials athletes.

Males (n = 9)					Females (n = 9)				
Name	Body form	Physiological function	Physical quality	Comprehensive index	Name	Body form	Physiological function	Physical quality	Comprehensive index
S**	Average	Average	Poor	Average	L**	Excellent	Good	Average	Average
L**	Average	Average	Average	Average	L**	Fail	Average	Poor	Fail
Y**	Poor	Average	Average	Average	J**	Average	Fail	Average	Poor
L**	Average	Poor	Fail	Fail	Y**	Poor	Average	Average	Average
F**	Fail	Average	Average	Poor	D**	Good	Average	Average	Average
S**	Excellent	Fail	Average	Average	S**	Average	Poor	Average	Average
L**	Average	Excellent	Average	Average	Z**	Excellent	Fail	Poor	Average
G**	Average	Fail	Average	Poor	Y**	Poor	Average	Average	Average
L**	Average	Average	Poor	Average	L**	Poor	Poor	Fail	Fail

#### Verification of evaluation standards

To further confirm the validity of the evaluation system, a frequency analysis on the comprehensive physical fitness rating results for male and female athletes from both elite and excellent groups was conducted (see Table 14). Based on the statistical results, the Fisher’s exact test was applied for data analysis. The test results showed that the p-values of the Fisher’s exact test for different groups of male and female athletes were all less than 0.001, indicating very significant differences between different groups. This not only verifies that the physical fitness level of athletes in the elite group is indeed better than that of the athletes in the excellent group, but also confirms that the high-level athlete physical fitness evaluation system constructed in this study can better reflect the physical fitness level of the athletes. Therefore, it is feasible to apply it to the physical fitness evaluation of high-level freestyle skiing aerials athletes.

**Table 14:** Frequency distribution of comprehensive physical fitness ratings for athletes in different groups.

	Male		Female	
Rating	Elite Group	Excellent Group	Elite Group	Excellent Group
Excellent	2		1	
Good	3		3	
Average		6	1	6
Poor	1	2		1
Fail		1		2
Total	6	9	5	9

To validate the stability and reliability of the physical fitness evaluation system, we selected athletes from the national training team for Freestyle Skiing Aerials preparing for the 25th Milan Winter Olympics as subjects of the study. This comprised 8 male and 8 female athletes. The average age of the male athletes was 20.13±2.17 years, with an average training duration of 9.81±3.48 years. For the female athletes, the average age was 21.45±4.07 years, with an average training duration of 10.94±2.93 years. All athletes participating in the tests have achieved rankings within the top three nationally. To ensure the accuracy of the testing, all athletes were uninjured before the tests and did not engage in high-intensity training the day before. A retest was conducted on the same group of athletes one week after the first test. After the completion of the testing, we scored the athletes’ physical fitness test data according to the scoring standards established in this study (see S7 Appendix). Kendall’s tau rank correlation was used to examine the comprehensive index scoring results of different groups. The results showed τ = 0.857 for male athletes and τ = 0.929 for female athletes, indicating that the evaluation system has good stability and reliability in the tests conducted at different points in time.

#### Construction of a physical fitness evaluation model for aerials athletes

The characteristic model of excellent athletes reflects the training target and can guide preselected objects from the actual state to the ideal state [52]. The special characteristics of freestyle skiing aerials indicate that physical fitness is one of the vital competitive abilities of these athletes. Through the quantitative description and theoretical induction of the selected typical index of the various elements of physical fitness, the physical fitness structure model of China’s high-level freestyle skiing aerials athletes was established. Its purpose is to provide guidance for the scientific selection and physical training planning and monitoring of Chinese freestyle skiing aerials athletes and improve the success rate of these high-level athletes. The research began with general and ideal models and constructed a special physical fitness model of China’s high-level freestyle skiing aerials athletes.

#### Construction of a general physical fitness model

This study established a general model of specific physical fitness based on the original data from the physical fitness test of 15 high-level male aerials athletes and 14 high-level female aerials athletes in China. The general model reflects the basic standard requirements for the physical fitness of excellent athletes in this sport and presents the commonness of the physical fitness characteristics of excellent athletes in this sport in the form of data quantification (see Table 15).

**Table 15:** General model of physical fitness of high-level freestyle skiing aerials athletes in China.

Structure of physical fitness	Content	Representative indexes	Basic feature	General model data (M±SD)	
				Male	Female
Body form	Body length	Achilles tendon length	1. Well-proportioned body 2. Narrow pelvis 3. Powerful strength of waist muscles	21.94±0.85	20.49±0.71
	Body width,	Pelvis width		26.90±1.55	28.25±5.80
	Circumference	Waist circumference		71.72±2.66	68.77±2.64
	Physical fullness	Quetelet index		393.99±20.57	347.19±24.79
Physiological function	Aerobic ability	Relative maximum anaerobic power	1. Strong anaerobic capacity 2. Good cardiopulmonary function 3. Strong oxygen operation ability	14.95±1.43	13.87±0.82
	Cardiopulmonary function	Relative maximum oxygen uptake		52.36±5.04	46.73±4.08
	Oxygen usage ability	Hemoglobin		151.60±7.67	127.14±7.51
Physical quality	Rapid strength of lower limbs	Power clean	1. Excellent rapid strength of lower limbs 2. Strong special core stability ability 3. Strong speed ability 4. Good aerobic endurance	87.90±19.85	73.43±13.25
	Muscle strength of the core body areas	Squat on the balance pad with the barbell raising		11.10±2.19	14.82±4.65
	Speed capability	30-meter sprint		4.42±0.72	4.98±0.40
	Aerobic endurance	12-minute run		2644±230.48	2435±272.53

#### Construction of the ideal physical fitness model

The general physical fitness model reflects the basic characteristics of high-level freestyle skiing aerials athletes but can only provide a basic reference range for developing athletes’ physical fitness indexes. This range fluctuates greatly and is not targeted. If athletes want to achieve excellent results in future international competitions (e.g., win or place second in the Olympic Games, World Championships, World Cup), we need to set a representative and symbolic goal for guidance in their physical training. Therefore, this paper takes the 90th percentile value of each index in the general model of high-level athletes as the reference value and establishes the ideal model of physical fitness for high-level freestyle skiing aerials athletes (see Table 16).

**Table 16:** Ideal model of physical fitness of high-level freestyle skiing aerials athletes in China.

Structure of physical fitness	Content	Representative indexes	Ideal model data (M±SD)	
			Male	Female
Body form	body length	Achilles tendon length	≥22.83	≥21.42
	body width,	Pelvis width	≥28.76	≥27.16
	circumference	Waist circumference	≥73.66	≥72.71
	Physical fullness	Quetelet index	≥424.72	≥373.86
Physiological function	Aerobic ability	Relative maximum anaerobic power	≥16.78	≥14.86
	Cardiopulmonary function	relative maximum oxygen uptake	≥50.20	≥51.06
	Oxygen usage ability	Hemoglobin	≥165	≥142
Physical quality	Rapid strength of lower limbs	Power clean	≥103	≥64
	Muscle strength of the core body areas	Squat on the balance pad with the barbell raising	≤9.09	≤10.04
	Speed capability	30-meter sprint	≤3.84	≤4.63
	Aerobic endurance	12-minute run	≥2939	≥2771

## Discussion

### Analysis of the characteristics of physical fitness evaluation indexes of aerials athletes

#### Analysis of body form index characteristics

The body form is defined as the athletes’ physical characteristics displayed externally or internally. Different body form indexes will impact sports technique and competitive strategy [53–55]. Freestyle skiing aerials require that the athletes’ shape not only meet the aesthetic requirements but also the mechanical characteristics to perform aerials.

Pelvic width is an index reflecting the transverse diameter of the lower part of the trunk. The structure of the pelvis can influence the range of motion at the hip joint [56]. From the point of view of sports biomechanics, the pelvis’s width significantly impacts those sports with lower limb movement as the central part. An overly wide pelvis can increase the risk of injuries to the anterior cruciate ligament (ACL) in the knee [57]. The technical characteristics of freestyle skiing aerials determine that the stability and balance of the athlete’s body and the control of the speed of skiing depend more on the lower limbs to compete better. A narrower pelvis can enhance movement efficiency [45, 58]. The purpose of selecting the index of pelvis width is to select athletes with an appropriate proportion of pelvis width. An appropriate pelvic width is conducive to developing athletes’ trunk rotation ability, adapting to the technical characteristics of aerial skills, and better reflecting the needs of beauty in the athletes’ form[46, 47].

Waist circumference mainly reflects the development level of athletes’ abdominal fat and muscle [36]. The selection of the waist circumference index reflects that freestyle skiing aerials have specific requirements for athletes’ waist strength. Athletes in this sport undergo substantial waist load during daily technical training. Systematic strength training can enhance waist muscle strength, thereby alleviating waist pressure [27]. In addition, freestyle skiing aerials have strict requirements for athletes’ core strength, especially in the lumbar and abdominal regions. On the one hand, when completing technical movements in the air, athletes should have better waist and abdomen strength to control the body to maintain the correct completion of the stunt [59]. On the other hand, during the landing phase, the strong neural control ability of the core muscles can reduce the risk of knee joint injury and improve landing performance [60, 61]. Therefore, if athletes want to maintain balance and stability, the correct position and completion of an action in the air and have a stable landing posture, strong waist and abdominal muscles are essential [15].

The Quetelet Index refers to the ratio of weight to height, which can reflect the development degree and symmetry of the human body form and can indirectly evaluate the muscle quality of athletes [36]. Typically, the higher the Quetelet Index of athletes, the more developed their physique is, indicating a higher density of body tissue and representing a better degree of physical fullness. The inclusion of this index suggests that the sport of freestyle skiing aerials requires athletes engaged in training for this event to possess excellent muscle mass and strength, to meet the stringent demands on physical conditions imposed by the increasingly fierce competition [28, 32].

Achilles tendon length refers to the distance from the lower abdominal edge of the medial gastrocnemius muscle to the calcaneal tubercle. In the exercise process, passive lengthening of the Achilles tendon plays a role in buffering and storing energy. Some studies have confirmed that the long and elastic Achilles tendon can store more energy and increase centrifugal buffering [62, 63]. After the athletes have successfully completed the three stages of assist slide, take-off, and aerial flip, the strength of the lower limbs and the ability of the body to maintain balance and control are the key factors that affect successful and stable landing [4, 12]. The longer Achilles tendon improves the cushioning capacity of the lower limbs and provides a prerequisite for landing stability [64].

#### Analysis of physiological function index characteristics

The level of physiological function can reveal the stimulation degree of the training load on the body, the appropriate load stimulation can promote motivation, and excessive load stimulation will destroy the body’s steady state and affect the training effect [29]. Therefore, physiological function is the key index to evaluate the adaptability of athletes to training, which is of great significance to control the training load and scientifically adjust the body state [30].

The primary physiological function of hemoglobin is to transport oxygen and carbon dioxide and to buffer acidic substances [65]. Within a specific range, the higher the hemoglobin content, the more oxygen it combines, and the higher the degree of bearing a high-intensity load [31]. Under high-intensity exercise, the body’s ability to resist fatigue is relatively strong, and thus, aerobic metabolism is also strong [30, 66]. Including this index indicates that freestyle skiing aerials have higher requirements for the aerobic metabolism of athletes. The interval between the technical movements of each jump of aerials athletes is more than 5 minutes, and the average duration of each competition or training is more than 120–240 minutes [26]. This requires the body to have a strong anti-fatigue ability, and the muscles need to have the ability to work for a long time. Therefore, athletes must have a strong aerobic metabolism ability to promote the recovery of energy materials, reduce muscle fatigue, and quickly recover physical fitness [67, 68].

The relative anaerobic power index reflects limb muscles’ ability to produce high mechanical power in a short time. It can be used to evaluate the energy supply capacity of the ATP-CP energy system [33]. This index is measured in terms of the number of watts produced per kilogram of body weight, directly reflecting the athlete’s limit of instantaneous force output in an anaerobic state. Freestyle skiing aerials are predominantly an anaerobic energy supply sport [69], which is particularly evident during phases like takeoff, somersault in the air, and landing. A higher value of relative maximum anaerobic power signifies that the athlete possesses stronger explosive power at critical moments, enabling them to perform more complex and high-difficulty maneuvers.

The relative maximum oxygen uptake directly reflects the level of human heart and lung function [70, 71]. Aerials athletes must perform many movements in training and competition, such as sliding, somersault, landing, and buffering. After each jump, athletes need to adjust their heart rate in time to prepare for the next jump. Due to the unique sports mode of the project, aerials athletes must have excellent cardiorespiratory function, which is also a prerequisite for athletes who bear heavy load training [32, 72].

#### Analysis of physical quality index characteristics

Physical quality is the most intuitive index to reflect the level of physical fitness. For aerials athletes, physical quality is the basis for acquiring the main content of complex and difficult skills and is also the key to ensuring the quality of technical movements in training and competition.

Power clean is an explosive training action, involving the kinetic chain of the entire body. Through the reaction force of the lower limbs, the power is transmitted to the waist and crotch and then to the upper limbs [73]. Finally, the weight of the barbell is carried through lower limb centrifugal buffering. When athletes perform this action, the muscles of the whole body work together. Therefore, this action is an internationally recognized explosive action [74, 75]. The starting sequence and transmission mode of the power chain of power clean match the power chain mode of take-off and landing in freestyle skiing aerials. It conforms to the movement characteristics of aerials athletes in multijoint and multimuscle group activities and can reflect the athletes’ overall strength level and coordination.

Squat on the balance pad with the barbell raising reflects the stability and strength of the athletes in the core area in the unsteady state and can also test the whole body’s flexibility. Athletes with strong special technical abilities can complete this action relatively quickly and in a relatively stable state [25]. The core stabilizing force plays a stabilizing and supporting role in the body posture, sports skills, and special technical actions of freestyle skiing aerials athletes [59]. It is an essential link for athletes to keep their body’s center of gravity in the correct position when completing technical actions. Completing technical action cannot be achieved by relying on a single muscle group alone. It must mobilize many muscle groups to work in coordination. The core muscle group plays the role of stabilizing the center of gravity, transmitting power, buffering, and so on in this process. It is also the main link of overall power generation and plays a pivotal role in connecting the upper and lower limbs to work together [76, 77]. The landing link of freestyle skiing aerials is crucial for athletes. After completing various movements in the air, athletes should stably land on slippery and sloping snow. Hence, the stability of the core area at the moment of landing is vital [15].

The 30-meter sprint mainly reflects the fast response and fast starting ability of athletes. It is also the comprehensive performance of speed, coordination, accuracy, flexibility, and other factors and sports skills in the process of sports. The smaller this index is, the faster the athlete reacts and moves. The technical actions of aerials require athletes to have the ability to respond quickly and take quick action [10]. The fast, accurate, and stable movement rhythm guarantees the smooth completion of high-quality technical movements, and the speed can lay a solid foundation for victory in a competition [78]. Thus, coaches must attach great importance to the speed and quality training of athletes to improve their overall performance levels.

A 12-minute run is a typical index of aerobic endurance level. Aerobic endurance refers to the ability of the body to maintain a particular intensity load and exercise for a long time within a specified time and with sufficient oxygen supply [35]. Freestyle skiing aerials is a high-intensity, short-duration sports event that relies on aerobic capacity as a basis to maintain overall endurance and recovery, and emphasizes the decisive role of anaerobic capacity at critical moments, such as providing explosive power in a short period [69]. During intervals or waiting periods in competitions or training, the aerobic oxidation system primarily functions to help rapidly restore energy in the body, especially replenishing the energy supply to the phosphagen system, thereby preparing athletes for high-intensity performances in the next phase [26]. Therefore, athletes should pay close attention to aerobic endurance training.

### Analysis of the current situation of the physical fitness level of aerials athletes

According to Table 12, in the comprehensive score of physical fitness, 2 men and women athletes in the first line are at a good level, and 2 are at an excellent level. Among the male athletes, Q * *’s first-level physical fitness index and comprehensive physical fitness evaluation are excellent. It shows that Q * * has outstanding physical fitness, which paves the way for him to better show his special technical ability. This athlete won the men’s freestyle skiing aerials individual competition at the 2022 Beijing Winter Olympic Games and the world championship title in this sport. Among the female athletes, X * *’s physical fitness level I index and comprehensive physical fitness evaluation are excellent, indicating her physical fitness level. This athlete won the gold medal in the women’s freestyle aerials individual competition at the 2022 Beijing Winter Olympic Games and made a breakthrough in the “zero” women’s gold medals in this sport in competitions in China. Currently, she is also the only Grand Slam winner in freestyle aerials. The above situation shows that this paper’s physical fitness evaluation system can better reflect the physical fitness level of excellent athletes in this sport. The evaluation results are objective. It is feasible to apply the physical fitness evaluation system to select athletes and evaluate physical fitness levels.

According to the rating results (Table 13), the physical fitness level of the national team’s second-tier freestyle skiing aerials athletes needs to be improved. In addition to the good physical fitness level of individual athletes and the balanced development of various elements of physical fitness, there are obvious areas for improvement in the physical fitness structure of most athletes. In the comprehensive rating, there are currently no athletes with good physical fitness or above among the national second-tier male and female athletes.

Through interviews with coaches, we learned that after the Beijing Winter Olympic Games, China’s freestyle skiing aerials team faced a transition between the old and the new. Due to the COVID-19 pandemic and other factors, there is a large gap between the current competitive ability of reserve talent and the best front-line athletes already in competition. The test object of this study is first-tier and second-tier freestyle skiing aerials athletes who were preparing for the 2022 Winter Olympics. The second-tier athletes are cross-event athletes who were selected five years ago and will also become first-tier athletes preparing for the 2026 Milan Winter Olympics. Therefore, in future training planning, coaches should develop personalized training programs for the weak links in the physical fitness levels of second-tier athletes to improve their physical fitness. It will pave the way for these freestyle aerials athletes to achieve better results in the next Winter Olympic Games.

### Analysis of the physical fitness evaluation model of aerials athletes

The general model of physical fitness is a model that represents the overall characteristics of the physical fitness of excellent athletes. It is the basic condition for entering the ranks of excellent athletes and a full summary of the structural characteristics of the physical fitness model of excellent athletes.

This study compares the test data of the national team’s first- and second-tier male and female athletes with the data of the ideal physical fitness model. At present, only the index data of the two champion athletes of the 2022 Beijing Winter Olympic Games have reached the ideal state, and the physical fitness of the rest of the athletes, including many athletes who have won the World Cup, is less than ideal. Therefore, this paper establishes an "ideal model" based on the 90th percentile value, which can provide some reference for future athletes to compensate for their physical deficiencies.

### Limitations and future outlook

This study constructed a physical fitness assessment system based on the test results of high-level athletes. Given that high-level athletes belong to a minority group, the sample size is limited and individual differences are present, which inevitably lead to occasional discrepancies in some data, affecting the universality of the assessment standards. Therefore, it is recommended that future research continues to expand the sample size of high-level athletes and optimize evaluation indexes to further improve and refine the assessment standards. Moreover, this study was unable to acquire relevant data from foreign athletes for a cross-sectional comparison, which constitutes a limitation of the current research. It is hoped that this aspect can be further supplemented and perfected in subsequent studies.

The physical fitness model of high-level athletes built in this study is not permanent. With the change in the rules of freestyle skiing aerials, the improvement of competition difficulty, and the enhancement of athletes’ competitive ability, the physical fitness model of athletes also needs constant correction and adjustment.

## Conclusion

### Establishment of a physical fitness evaluation system for freestyle skiing aerialists

This study constructs an evaluation system for the physical fitness of freestyle skiing aerialists in China, including evaluation indexes, index weights, and evaluation criteria. The evaluation indexes include 3 first-level indexes, 11 second-level indexes, and 11 third-level indexes in terms of body form, physiological function, and physical quality. In the evaluation, the weight of physical quality accounted for the largest weight, and physiological function and body form ranked second and third, respectively. The evaluation criteria consist of scoring evaluation criteria and rating evaluation criteria. The test results of the athletes verify that the evaluation system is objectively effective.

### Establishment of physical fitness model for freestyle skiing aerialists

Based on the index system, this study constructs a general and ideal physical fitness model of high-level freestyle skiers in China, providing reference and guidance for athletes’ training and evaluation, monitoring and diagnosis, and scientific selection.

### Recommendation

When the evaluation system constructed by this research institute is applied to sports teams, the required equipment for physical quality and body form testing indexes are easily obtained, and the funding cost is not high. However, physiological function testing equipment is relatively expensive, and it is recommended to obtain it from university sports research centers or sports research and training bases.

During the index test, it is suggested that the test of morphological and physiological indexes should be carried out by sports scientific researchers of relevant majors (human sports science, etc.). The physical quality test is conducted by professionals with relevant qualifications as physical fitness coaches. It is recommended that two professionals be responsible for the testing content of one athlete. In addition, it is recommended to add 1–2 camera personnel during testing to prevent any disputes during testing from being available for review.

## Supporting information

S1 Appendix Expert Interview outline. (PDF)

S2 Appendix Index survey questionnaire and results. (PDF)

S3 Appendix Index weight questionnaire and results. (PDF)

S4 Appendix Questionnaire validity assessment and results. (PDF)

S5 Appendix Detailed rules for physical fitness index testing. (PDF)

S6 Appendix Data analysis details. (PDF)

S7 Appendix Retest scoring results of physical fitness indexes. (PDF)
