# Supplementary material for: Construction of a physical fitness evaluation index system and model for high-level freestyle skiing aerials athletes in China
Source: PLoS One. 2023 Dec 8;18(12):e0295622. doi: 10.1371/journal.pone.0295622 (PMC10707543; doi:10.1371/journal.pone.0295622)
Supplement: S3 Appendix — (PDF) [file pone.0295622.s003.pdf]

Appendix 3-1

Expert questionnaire of weight coefficient of physical fitness evaluation index of Chinese high-level Freestyle Skiing Aerials athletes

Respected Expert,

Greetings!

Thank you immensely for taking the time out of your busy schedule to complete this questionnaire. This survey is a crucial component of our research project titled, “Construction of a physical fitness evaluation index system and model for high-level Freestyle Skiing Aerials athletes in China.” This study aims to conduct a comprehensive assessment of the physical fitness of high-level athletes from the national training team for freestyle skiing aerials preparing for the Beijing Winter Olympics. The ultimate goal is to provide insights for the scientific training of Chinese freestyle skiing aerials athletes and to enhance the quality of their physical fitness training.

The primary objective of this questionnaire is to determine the weights of indexes related to body form physiological function, and physical quality. Your insights and expertise are invaluable to this research. We genuinely hope for your support and understanding and would be profoundly grateful if you could assist by completing this survey. Please rest assured that all information provided will remain strictly confidential and will be used solely for the purposes of this research.

Once again, I express my sincere gratitude for your invaluable assistance.

1.Your basic information

Name：	
Age:	
Gender:	
Affiliation:	
Position:	
Professional title:	
Educational level:	
Research area:	
Years of teaching or Training experience:	

2.Score table

Below are the representative indexes reflecting the physical fitness evaluation system for freestyle skiing aerials athletes. Based on the relevance of each index to the specialized physical fitness of freestyle skiing aerials athletes, please assign an appropriate score. The scores are divided into five levels: Very important = 5 points, Quite important = 4 points, Neutral = 3 points, Not important = 2 points, Very unimportant = 1 point. Simply mark “ √ ” under the corresponding score.

2.1 Weighted Scoring for male athlete indexes

Table 1 Weighted scoring table of first-level physical fitness indexes

First-level index	Very important	Quite important	Neutral	Not important	Very unimportant
Body form					
Physiological function					
Physical quality					

Table 2 Weighted scoring table of second-level and third-level physical fitness indexes

First-level index	Second-level index	Third-level index	Very important	Quite important	Neutral	Not important	Very unimportant
Body form	Body length	Achilles tendon length					
	Body width	Pelvis width					
	Body circumference	Waist circumference					
	Body composition	Quetelet index					
Physiological function	Aerobic ability	Relative maximum anaerobic power					
	Cardiopulmonary performance	Relative maximum oxygen uptake					
	Exercise biochemical ability	Hemoglobin					
Physical quality	Limb strength	Power clean					
	Core strength	Squat on the balance pad with the barbell raising					
	Speed-agility	30-meter sprint					
	Aerobic capacity	12-minute run					

2.2 Weighted Scoring for female athlete indexes

Table 3 Weighted scoring table of first-level physical fitness indexes

First-level index	Very important	Quite important	Neutral	Not important	Very unimportant
Body form					
Physiological function					
Physical quality					

Table 4 Weighted scoring table of second-level and third-level physical fitness indexes

First-level index	Second-level index	Third-level index	Very important	Quite important	Neutral	Not important	Very unimportant
Body form	Body length	Achilles tendon length					
	Body width	Pelvis width					
	Body circumference	Waist circumference					
	Body composition	Quetelet index					
Physiological function	Aerobic ability	Relative maximum anaerobic power					
	Cardiopulmonary performance	Relative maximum oxygen uptake					
	Exercise biochemical ability	Hemoglobin					
Physical quality	Limb strength	Power clean					
	Core strength	Squat on the balance pad with the barbell raising					
	Speed-agility	30-meter sprint					
	Aerobic capacity	12-minute run					

If you have any additional comments or suggestions, please fill in here:

Once again, we sincerely thank you for your strong support!

Appendix 3-2

1. Results of the index weight survey of male athletes

Table 1 Survey results of first-level physical fitness index weight (n=14)

First-level index	Very important	Quite important	Neutral	Not important	Very unimportant
Body form		**3**	**8**	**3**	
Physiological function	**1**	**11**	**2**		
Physical quality	**13**	**1**			

Table 2 Results of second-level and third-level physical fitness index weight (n=14)

First-level index	Second-level index	Third-level index	Very important	Quite important	Neutral	Not important	Very unimportant
Body form	Body length	Achilles tendon length	**1**	**5**	**6**	**2**	
	Body width	Pelvis width		**4**	**10**		
	Body circumference	Waist circumference	**5**	**9**			
	Body composition	Quetelet index		**4**	**7**	**3**	
Physiological function	Aerobic ability	Relative maximum anaerobic power	**11**	**3**			
	cardiopulmonary performance	Relative maximum oxygen uptake	**6**	**6**	**2**		
	Exercise biochemical ability	Hemoglobin	**9**	**5**			
Physical quality	Limb strength	Power clean	**12**	**2**			
	Core strength	Squat on the balance pad with the barbell raising	**9**	**5**			
	Speed-agility	30-meter sprint	**1**	**5**	**5**	**3**	
	Aerobic capacity	12-minute run	**3**	**5**	**2**	**4**	

2. Results of the index weight survey of female athletes

Table 1 Survey Results of first-level physical fitness index weight (n=14)

First-level index	Very important	Quite important	Neutral	Not important	Very unimportant
Body form		**4**	**10**		
Physiological function	**2**	**8**	**4**		
Physical quality	**13**	**1**			

Table 2 Results of second-level and third-level physical fitness index weight (n=14)

First-level index	Second-level index	Third-level index	Very important	Quite important	Neutral	Not important	Very unimportant
Body form	Body length	Achilles tendon length		**5**	**9**		
	Body width	Pelvis width		**3**	**11**		
	Body circumference	Waist circumference	**6**	**8**			
	Body composition	Quetelet index	**1**	**8**	**3**	**2**	
Physiological function	Aerobic ability	Relative maximum anaerobic power	**11**	**3**			
	cardiopulmonary performance	Relative maximum oxygen uptake	**2**	**9**	**3**		
	Exercise biochemical ability	Hemoglobin	**10**	**4**			
Physical quality	Limb strength	Power clean	**9**	**5**			
	Core strength	Squat on the balance pad with the barbell raising	**11**	**3**			
	Speed-agility	30-meter sprint		**7**	**7**		
	Aerobic capacity	12-minute run	**4**	**9**	**1**		

Appendix 3-3

Expert evaluation table of the weight coefficient of physical fitness evaluation index of Chinese high-level Freestyle Skiing Aerials athletes

Based on the expert weight questionnaire survey (using a 1-5 point scoring system), we applied a normalization process to calculate the weight coefficients of various physical fitness indexes for freestyle skiing aerials athletes in China. Do you think these weight values are appropriate? (Please mark " √ " in the box if you agree.)

Table 1 Statistical table of the weight coefficient of first-level indexes

First-level index	**Weight (Male/Female)**	**Agree**	**Disagree**	**Suggested modifications**
A1 Body form	0.25/0.27			
B2 Physiological function	0.33/0.32			
C3 Physical quality	0.42/0.41			

Table 2 Statistical table of the weight coefficient of the second-level and third-level indexes

**First-level index**	**Second-level index**	**Third-level index**	**Weight (Male/Female)**	**Agree**	**Disagree**	**Suggested modifications**
A1 Body form	A1 Body length	A1 Achilles tendon length				
	A2 Body width	A2 Pelvis width				
	A3 Body circumference	A3 Waist circumference				
	A4 Body composition	A4 Quetelet index				
A2 Physiological function	B1 Aerobic ability	B1 Relative maximum anaerobic power				
	B2Cardiopulmonary performance	B2 Relative maximum oxygen uptake				
	B3 Exercise biochemical ability	B3 Hemoglobin				
A3 Physical quality	C1 Limb strength	C1 Power clean				
	C2 Core strength	C2 Squat on the balance pad with the barbell raising				
	C3 Speed-agility	C3 30-meter sprint				
	C4 Aerobic capacity	C4 12-minute run				

Appendix 3-4

Results of the expert evaluation on the weight coefficient of physical fitness assessment Index (n=6)

Table 1 Statistical table of the weight coefficient of first-level indexes

First-level index	Weight (Male/Female)	Agree	Disagree	Suggested modifications
A1 Body form	0.25/0.27	100%		
		100%		
		100%		
		100%		
B2 Physiological function	0.33/0.32	100%		
		100%		
		100%		
C3 Physical quality	0.42/0.41	100%		
		100%		
		100%		
		100%		

Table 2 Statistical table of the weight coefficient of the second-level and third-level indexes

First-level index	Second-level index	Third-level index	Weight (Male/Female)	Agree	Disagree	Suggested modifications
A1 Body form	A1 Body length	A1 Achilles tendon length	0.24/0.23	100%		
	A2 Body width	A2 Pelvis width	0.23/0.22	100%		
	A3 Body circumference	A3 Waist circumference	0.31/0.30	100%		
	A4 Body composition	A4 Quetelet index	0.22/0.25	100%		
A2 Physiological function	B1 Aerobic ability	B1 Relative maximum anaerobic power	0.35/0.36	100%		
	B2Cardiopulmonary performance	B2 Relative maximum oxygen uptake	0.31/0.29	83.33%	16.67%	
	B3 Exercise biochemical ability	B3 Hemoglobin	0.34/0.35	100%		
A3 Physical quality	C1 Limb strength	C1 Power clean	0.30/0.27	100%		
	C2 Core strength	C2 Squat on the balance pad with the barbell raising	0.29/0.28	100%		
	C3 Speed-agility	C3 30-meter sprint	0.20/0.20	100%		
	C4 Aerobic capacity	C4 12-minute run	0.21/0.25	100%		
