# Supplementary material for: Construction of a physical fitness evaluation index system and model for high-level freestyle skiing aerials athletes in China
Source: PLoS One. 2023 Dec 8;18(12):e0295622. doi: 10.1371/journal.pone.0295622 (PMC10707543; doi:10.1371/journal.pone.0295622)
Supplement: S4 Appendix — (PDF) [file pone.0295622.s004.pdf]

Appendix 4-1

Expert validity evaluation form for survey questionnaire

1. Basic Information:

Name:	Gender:
Professional title:	Position:
Field of study:	Affiliation:

2. Validity Test (Please mark “ √ ” in the appropriate box corresponding to your assessment level)

2.1 How do you evaluate the overall design of the questionnaire?

Very satisfied	Satisfied	Fairly satisfied	Dissatisfied	Very dissatisfied

2.2 How do you assess the structural design of the questionnaire?

Very satisfied	Satisfied	Fairly satisfied	Dissatisfied	Very dissatisfied

2.3 How do you rate the content design of the questionnaire?

Very satisfied	Satisfied	Fairly satisfied	Dissatisfied	Very dissatisfied

Appendix 4-2

1. Results of validity test for physical fitness evaluation index selection questionnaire (n=12)

1. Overall questionnaire design test results

	Very satisfied	Satisfied	Fairly satisfied	Dissatisfied	Very dissatisfied
Number	6	5	1		
Percentage	50.00%	41.67%	8.33%		

2. Questionnaire structure design test results

	Very satisfied	Satisfied	Fairly satisfied	Dissatisfied	Very dissatisfied
Number	5	7			
Percentage	41.67%	58.33%			

3. Questionnaire content design test results

	Very satisfied	Satisfied	Fairly satisfied	Dissatisfied	Very dissatisfied
Number	6	5	1		
Percentage	50.00%	41.67%	8.33%		

2. Results of validity test the physical fitness index weight questionnaire (n=6)

1.Overall questionnaire design test results

	Very satisfied	Satisfied	Fairly satisfied	Dissatisfied	Very dissatisfied
Number	1	5			
Percentage	16.67%	83.33%			

2. Questionnaire structure design test results

	Very satisfied	Satisfied	Fairly satisfied	Dissatisfied	Very dissatisfied
Number	2	4			
Percentage	33.33%	66.67%			

3.Questionnaire content design test results

	Very satisfied	Satisfied	Fairly satisfied	Dissatisfied	Very dissatisfied
Number	2	4			
Percentage	33.33%	66.67%			
