# Supplementary material for: Construction of a physical fitness evaluation index system and model for high-level freestyle skiing aerials athletes in China
Source: PLoS One. 2023 Dec 8;18(12):e0295622. doi: 10.1371/journal.pone.0295622 (PMC10707543; doi:10.1371/journal.pone.0295622)
Supplement: S5 Appendix — (PDF) [file pone.0295622.s005.pdf]

S5 Appendix Detailed rules for physical fitness index testing

1. Detailed rules for body form index testing

Index	Testing Equipment	Testing Method
Height	Standard height measuring instrument	(1) The athlete should stand barefoot in the upright position on the base of the stadiometer. The arms should hang naturally, the heels should be together, and the toes should be spread apart at 60 degrees. The heels, sacrum, and the area between the two scapulae should touch the stadiometer's pillar. The torso should be naturally upright, the head should be straight, and the eyes should look straight ahead. The athlete should maintain a horizontal position between the upper edge of the ear and the lower edge of the orbit.<br>(2) The tester stands to the right of the participant and gently slides the horizontal pressure plate down the pillar, lightly pressing it on top of the participant's head. When the tester reads the measurement, their eyes should be at the same height as the pressure plate. The measurement is recorded after the recorder repeats it.<br>(3) Measurements are taken in centimeters, accurate to one decimal place, and are filled into the grid. The testing error must not exceed 0.5 centimeters.
Upper limb length	(1) Length measuring instrument or tape measure.<br>(2) Prior to use, it should be calibrated with a standard steel ruler, with the error not exceeding 0.2 cm per meter.	(1) The athlete, barefoot, stands on a flat surface, with feet shoulder-width apart, maintaining a natural standing position.<br>(2) The tester stands on the left side of the athlete, and then uses a measuring tape to measure the distance from the athlete's acromion to the tip of the fingers.<br>(3) The tester reads out the measurements. At this time, the recorder repeats the measurements and records them in a notebook. (Measurements are in centimeters, accurate to one decimal place, and the measurement error must not exceed 0.2CM).
Lower limb length	(1) Vernier steel ruler<br>(2) Prior to use, it should be calibrated with a standard steel ruler, with the error not exceeding 0.2 cm per meter.	(1) The athlete, barefoot, stands on a flat surface, with feet shoulder-width apart, maintaining a natural standing position.<br>(2) The tester stands at the athlete's side, places the measuring tape vertically on the ground, and at the same time, locates the position of the greater trochanter of the femur and the tip of the medial malleolus, measuring their linear distance.<br>(3) The tester reads out the measurements. At this time, the recorder repeats the measurements and records them in a notebook.
Achilles tendon length	(1) Vernier steel ruler<br>(2) Prior to use, it should be calibrated with a standard steel ruler, with the error not exceeding 0.2 cm per meter.	(1) The athlete, barefoot, faces the wall, stands on a flat surface, with feet shoulder-width apart, maintaining a natural standing position. Then, the athlete performs a heel-raising exercise against the wall until the triceps surae is fully contracted.<br>(2) The tester marks a point on the medial edge of the belly of the athlete's gastrocnemius muscle, then allows the athlete to

		return to a natural standing position, feet shoulder-width apart, parallel stance, and then measures the vertical distance from the marked point to the flat surface.
Shoulder width	(1) Caliper<br>(2) Before use, it should be calibrated, with the error not exceeding 0.2 cm.	The tester stands naturally, the tester locates the acromion of the tester, and then uses a measuring tape to measure the shortest distance between the two bony landmarks, with measurements accurate to 0.1 cm.
Pelvis width	(1) Caliper<br>(2) Before use, it should be calibrated, with the error not exceeding 0.2 cm.	(1) The athlete, barefoot, stands on a flat surface, with feet shoulder-width apart, maintaining a natural standing position.<br>(2) The tester crouches in front of the athlete, touches the widest part of the spine, and then uses the designated measuring tape to measure the widest distance between the two iliac crests.
Thigh circumference	(1) Plastic tape measure lined with nylon thread.<br>(2) Before use, it must be calibrated with a steel tape measure, with the error not exceeding 0.2 cm per meter.	(1) The athlete, barefoot, stands on a flat surface, with feet shoulder-width apart, maintaining a natural standing position, and the tester wraps the measuring tape around the middle third of the thigh, measuring its circumference.<br>(2) The tester reads out the measurements, and the recorder repeats them and records them.
Calf circumference	(1) Plastic tape measure lined with nylon thread.<br>(2) Before use, it must be calibrated with a steel tape measure, with the error not exceeding 0.2 cm per meter.	(1) The athlete stands barefoot on a level ground, with feet standing as wide as the shoulders, maintaining a natural standing position.<br>(2) The measurer instructs the athlete to lift one foot onto a stool so that the angle between the thigh and the calf is 90 degrees. The measurer uses a tape measure to measure the thickest part of the athlete's calf, taking measurements in a horizontal circle around it.<br>(3)After measurement, the measurer reads out the number, and the recorder repeats it before recording it.
Waist circumference	(1) Plastic tape measure lined with nylon thread.<br>(2) Before use, it must be calibrated with a steel tape measure, with the error not exceeding 0.2 cm per meter.	(1) The athlete naturally stands with their feet shoulder-width apart, their waist and abdomen relaxed, and their breathing steady. The measurer wraps a tape measure around the narrowest part of the waist between the ribs and the iliac crest. The number where the tape intersects with zero is the waist circumference.<br>(2) The measurer reads out the number and the recorder repeats it and records the data in a notebook.
Body weight	(1) Specified electronic scale.<br>(2) The display of the electronic scale should be accurate to 0.1 kg.	(1) During the test, the digital weight scale should be placed on a flat surface and adjusted to zero. The athlete stands in shorts in the center of the scale until the digital weight scale reads a value.<br>(2) The recorder repeats the reading and fills it in the table. The measurement error should not exceed 0.1 kg.
Body composition	A multifrequency segmented bioelectrical impedance body composition tester (model: InBody270,	Measurements are strictly carried out according to the user manual and operation manual provided with the instrument.

	Yinbadi Co., Ltd., CN)	

2. Detailed rules for physiological function index testing

Index	Testing Equipment	Testing Method
Anaerobic capacity	One anaerobic power bicycle (model MONARK837, Switzerland)	Test method and steps:<br>(1) The participant wears sportswear and sneakers and adjusts the seat height and handle position of the ergometer according to their height. They pedal quickly and repeatedly without resistance for 1-3 minutes to familiarize themselves with the equipment, while the tester explains the test process and requirements.<br>(2) The participant is required to do warm-up exercises for 3-5 minutes, including 2-3 sprint practices, to reach a fully mobilized state of muscles and organ systems. At the same time, the load in the code removal basket is adjusted to the specified value according to 0.075kg/kg body weight.<br>(3) When the heart rate recovers to 100 beats/min, the participant tightens the pedals to prepare for pedaling. The tester gives the start command, the participant starts pedaling at full power under no-load conditions. When the participant quickly accelerates to over 180 revolutions/min, the tester pulls down the code removal basket to load the resistance and simultaneously clicks the StartTimer button. The participant pedals at full power for 30 seconds continuously, and the tester continues to provide verbal encouragement until the participant pedals for 30 seconds to the best of their ability.

Oxygen usage ability	(1) One exercise cardiopulmonary function test system (model MAXII, USA)<br>(2) One bicycle ergometer（model：Monark839E，Switzerland）	**Test procedure and method:**<br>(1) First, a general physical examination is carried out, and then the significance and requirements of the experiment are explained to encourage the athlete's active cooperation.<br>(2) Before the maximum oxygen intake test begins, the seat height of the ergometer is adjusted, a face mask is worn, and any air leaks and discomfort are checked.<br>(3) The receiver of the wristwatch-style heart rate telemetry device is attached to the sternum and the apex of the heart to ensure accurate heart rate measurement. Warm-up for 5 minutes, heart rate 140-150 beats/min, rest for 3 minutes before starting the test.<br>(4) After the test ends, the athlete is asked to pedal for another 5 minutes at a load of 50W for recovery rest.<br><br>**The criteria for judging maximum oxygen uptake:**<br>(1) Respiratory quotient above 1.10<br>(2) Heart rate reaches the individual's maximum heart rate<br>(3) Despite the increase in exercise load, VO2 remains stable or the difference between two oxygen intake is less than 5% (below 150ml per minute or 2ml/kg/min)<br>The participant is exhausted and cannot maintain a specific intensity of exercise.
Hemoglobin	One hemoglbin analyzer (model: XF-IB, CN)	Finger blood is collected for testing under quiet conditions in the early morning.
Red-cell count	One red blood cell analyzer (model: BECKMAN STKS, CN)	Finger blood is collected for testing under quiet conditions in the early morning.
Blood urea	One blood urea semiautomatic biochemical analyzer (model: BT-1904C, CN)	Finger blood is collected for testing under quiet conditions in the early morning.
Serum testosterone	One serum testosterone analyzer (model: DSL-10-4000, USA).	Venous blood is collected for testing under quiet conditions in the early morning.
Serum cortisol	One cortisol analyzer (model: DSL-10-67100, USA)	Venous blood is collected for testing under quiet conditions in the early morning.

3. Detailed rules for physical quality index testing

Index	Testing Equipment	Testing Method
Squat up	(1) Squat rack (Zhang Kong barbell Manufacturing Co., Ltd., CN)<br>(2) Barbell bars (Zhang Kong barbell Manufacturing Co., Ltd., CN)<br>(3) Barbell tablets (Zhang Kong barbell Manufacturing Co., Ltd., CN)	(1) At the start, the athlete places the barbell on the upper part of the posterior deltoids, grasping it with both hands slightly wider than shoulder-width apart. Feet are positioned a little wider than the shoulders, with toes slightly outward.<br>(2) Then, the athlete begins to execute the squat.

		(3) For the athlete's safety, there must be two spotters on either side of the barbell.
Pull up	Ribbed wooden frame (WaterRower Co., Ltd., Germany)	(1) The starting position has the athlete firmly grasping a pull-up bar with arms fully extended and torso hanging.<br>(2) The athlete then pulls up their body until their chin surpasses the bar, minimizing excessive body swinging.<br>(3) Swiftly returning to the starting position, they perform another pull-up, and continue until exhaustion.
Power clean	(1) barbell bars (Zhang Kong barbell Manufacturing Co., Ltd., CN)<br>(2) barbell tablets (Zhang Kong barbell Manufacturing Co., Ltd., CN)	(1) The athlete stands with feet shoulder-width apart, legs close to the barbell, and hands gripping slightly wider than the shoulders; the athlete squats until the upper thighs are parallel to the ground, keeping the back straight, body approximately at a 45-degree angle to the ground.<br>(2) Shoulders locked, the three lower joints (hip, knee, ankle) exert force to lift and flip the barbell to chest level, holding for two seconds.
Standing long jump	(1) White tape<br>(2) Tape (Great Wall seiko Industrial Co., Ltd., CN)	The athlete stands in front of the jump line (toes touching the line), with feet shoulder-width apart. They swing their arms rapidly and jump forward as far as possible.
Side throw (left and right)	(1) 10 lb，8 lb hard medicine balls (Jia You sports and leisure products Co., Ltd., CN)<br>(2) White tape<br>(3) Tape (Great Wall seiko Industrial Co., Ltd., CN)<br>(4) Yoga mats (Jia You sports and leisure products Co., Ltd., CN)	(1)The athlete sits sideways on a yoga mat, with both hands holding a medicine ball on the opposite side, positioned at the waist.<br>(2) When throwing the ball, there should be no body swinging. The ball is thrown quickly with a twist of the body, and the body remains stable after the ball is released.<br>(3) The weight of the medicine ball is 10 pounds for male athletes and 8 pounds for female athletes.
Back throw	(1) 10 lb，8 lb hard medicine balls (Jia You sports and leisure products Co., Ltd., CN)<br>(2) White tape<br>(3) Tape (Great Wall seiko Industrial Co., Ltd., CN)<br>(4) Yoga mats (Jia You sports and leisure products Co., Ltd., CN)	(1) Athletes kneel with their back facing the direction of the throw, holding the ball in front of their body with both hands.<br>(2) When throwing, the body must not swing; the ball is thrown backwards with a hip extension and double-arm throw, and the body remains stable after the ball is released.<br>(3) The weight of the backward thrown medicine ball is 10 pounds for male athletes and 8 pounds for female athletes.
Single-leg triple jump (left and right)	(1) Ground track field<br>(2) Tape (Great Wall seiko Industrial Co., Ltd., CN)<br>(3) White tape	(1) The athlete stands with feet apart in a jumping position, aiming to leap as far as possible. The jump begins with a single foot (left/right) landing, and after stabilizing, the athlete proceeds to jump forward. They do three consecutive jumps, finishing with a two-foot landing.<br>(2) The measuring staff measure the jumping distance of the athlete.<br>(3) The test is performed three times, taking the longest stable jump distance as the final score.

Squat on the balance pad with the barbell raising	(1) Balance pads (Jia You sports and leisure products Co., Ltd., CN)<br>(2) 20KG, barbell bars (Zhang Kong barbell Manufacturing Co., Ltd., CN)<br>(3) Barbell tablets (Zhang Kong barbell Manufacturing Co., Ltd., CN)<br>(4) Arbell buckle(Zhang Kong barbell Manufacturing Co., Ltd., CN)<br>(5) Electronic stopwatches (SEIKO, SVAJ007, Japan)	(1) The athlete raises a barbell straight over their head with both hands. The weight of the barbell is 40KG for male athletes and 30KG for female athletes.<br>(2) They stand on two balance plates with their feet hip-width apart, squatting quickly until their thighs are parallel to the ground.<br>(3) They quickly complete 10 repetitions in succession, recording the best score out of three attempts.
Quick v-up （15 repetitions）	(1) Yoga mats (Jia You sports and leisure products Co., Ltd., CN)<br>(2) Electronic stopwatches (SEIKO, SVAJ007, Japan)	(1) Starting position: The hands are placed by the ears, the legs are extended, and the feet are hooked. Each time, all four limbs touch the ground.<br>(2) Test process: The athlete's waist and abdomen contract rapidly. After the hands touch the feet, they return to the starting position and quickly complete 15 repetitions. The coach records the fastest speed.
30-meter sprint	(1) Standard plastic runway<br>(2) An optical door timing system (SmartSpeed, Fusion Sport Ltd., Australia)<br>(3) White tape	The athlete begins at the starting line with a standing start, sprinting rapidly forward for 30 meters until crossing the finish line. The measurement data is accurate to two decimal places.
12-minute run	(1) Ground track field<br>(2)Electronic stopwatches (SEIKO, SVAJ007, Japan)	The athlete begins at the starting line in a standing start position. Upon hearing the starting gun, they set off from the starting line and continue running for 12 minutes. The coach records the distance the athlete completes.
Agile running	(1) Ground track field、logo discs (Jia You sports and leisure products Co., Ltd., CN)<br>(2) Tape (Great Wall seiko Industrial Co., Ltd., CN)<br>(3) Electronic stopwatches (SEIKO, SVAJ007, Japan)	The athlete starts from one side, accelerates to the second marker, then runs in reverse towards the first marker between the first and second on the left side. After weaving through three markers in a serpentine pattern, they accelerate back to the starting point. The fastest speed is tested.
