# Supplementary material for: Construction of a physical fitness evaluation index system and model for high-level freestyle skiing aerials athletes in China
Source: PLoS One. 2023 Dec 8;18(12):e0295622. doi: 10.1371/journal.pone.0295622 (PMC10707543; doi:10.1371/journal.pone.0295622)
Supplement: S6 Appendix — (PDF) [file pone.0295622.s006.pdf]

S2 Appendix

1 Body form

1.1 Body form of male athletes

1.1.1 Cluster analysis

Table 1 Proximity matrix for clustering analysis of male athletes’ body form

Proximity Matrix															
**Case**	Height	Body weight	Upper limb length	Lower limb length	Achilles tendon length	Shoulder width	Pelvis wide	Thigh circumference	Calf circumference	Waist circumference	Fat-free body weight	Body fat percentage	Waist-hip ratio	Body mass index	Quetelet index
Height	1.000	0.397	0.446	0.462	0.108	-0.403	-0.008	-0.803	-0.455	-0.581	0.167	0.412	0.103	0.278	0.365
Body weight	0.397	1.000	0.320	0.262	0.108	-0.250	0.279	-0.269	0.221	0.331	0.797	0.564	0.495	0.767	0.948
Upper limb length	0.446	0.320	1.000	0.938	0.640	-0.551	-0.331	-0.338	0.251	-0.217	0.163	0.275	0.433	0.100	0.229
Lower limb length	0.462	0.262	0.938	1.000	0.538	-0.566	-0.240	-0.392	0.206	-0.227	0.109	0.250	0.341	0.005	0.151
Achilles tendon length	0.108	0.108	0.640	0.538	1.000	-0.197	-0.510	-0.035	0.405	-0.093	0.010	0.141	0.304	0.134	0.125
Shoulder width	-0.403	-0.250	-0.551	-0.566	-0.197	1.000	0.469	0.285	0.240	0.273	0.143	-0.585	-0.543	0.066	-0.106
Pelvis wide	-0.008	0.279	-0.331	-0.240	-0.510	0.469	1.000	-0.282	0.218	0.241	0.396	-0.059	-0.386	0.401	0.362
Thigh circumference	-0.803	-0.269	-0.338	-0.392	-0.035	0.285	-0.282	1.000	0.242	0.475	-0.065	-0.354	0.136	-0.169	-0.238
Calf circumference	-0.455	0.221	0.251	0.206	0.405	0.240	0.218	0.242	1.000	0.608	0.386	-0.156	0.074	0.060	0.154
Waist circumference	-0.581	0.331	-0.217	-0.227	-0.093	0.273	0.241	0.475	0.608	1.000	0.385	0.037	0.136	0.153	0.266
Fat-free body weight	0.167	0.797	0.163	0.109	0.010	0.143	0.396	-0.065	0.386	0.385	1.000	-0.048	0.070	0.593	0.744
Body fat percentage	0.412	0.564	0.275	0.250	0.141	-0.585	-0.059	-0.354	-0.156	0.037	-0.048	1.000	0.723	0.455	0.549
Waist-hip ratio	0.103	0.495	0.433	0.341	0.304	-0.543	-0.386	0.136	0.074	0.136	0.070	0.723	1.000	0.316	0.437
Body mass index	0.278	0.767	0.100	0.005	0.134	0.066	0.401	-0.169	0.060	0.153	0.593	0.455	0.316	1.000	0.931
Quetelet index	0.365	0.948	0.229	0.151	0.125	-0.106	0.362	-0.238	0.154	0.266	0.744	0.549	0.437	0.931	1.000

Table 2 Agglomeration schedule for clustering analysis of male athletes' body form

Agglomeration Schedule						
Stage	Cluster Combined		Coefficients	Stage Cluster First Appears		Next Stage
	Cluster 1	Cluster 2		Cluster 1	Cluster 2	
1	2	15	.948	0	0	3
2	3	4	.938	0	0	7
3	2	14	.849	1	0	5
4	12	13	.723	0	0	10
5	2	11	.711	3	0	10
6	9	10	.608	0	0	9
7	3	5	.589	2	0	11
8	6	7	.469	0	0	13
9	8	9	.359	0	6	13
10	2	12	.355	5	4	12
11	1	3	.339	0	7	12
12	1	2	.216	11	10	14
13	6	8	.162	8	9	14
14	1	6	-.086	12	13	0

Table 3 Cluster membership of male athletes' body form

Cluster Membership	
Case	4 Clusters
Height	1
Body weight	2
Upper limb length	1
Lower limb length	1
Achilles tendon length	1
Shoulder width	3
Pelvis wide	3
Thigh circumference	4
Calf circumference	4
Waist circumference	4
Fat-free body weight	2
Body fat percentage	2
Waist-hip ratio	2
Body mass index	2
Quetelet index	2

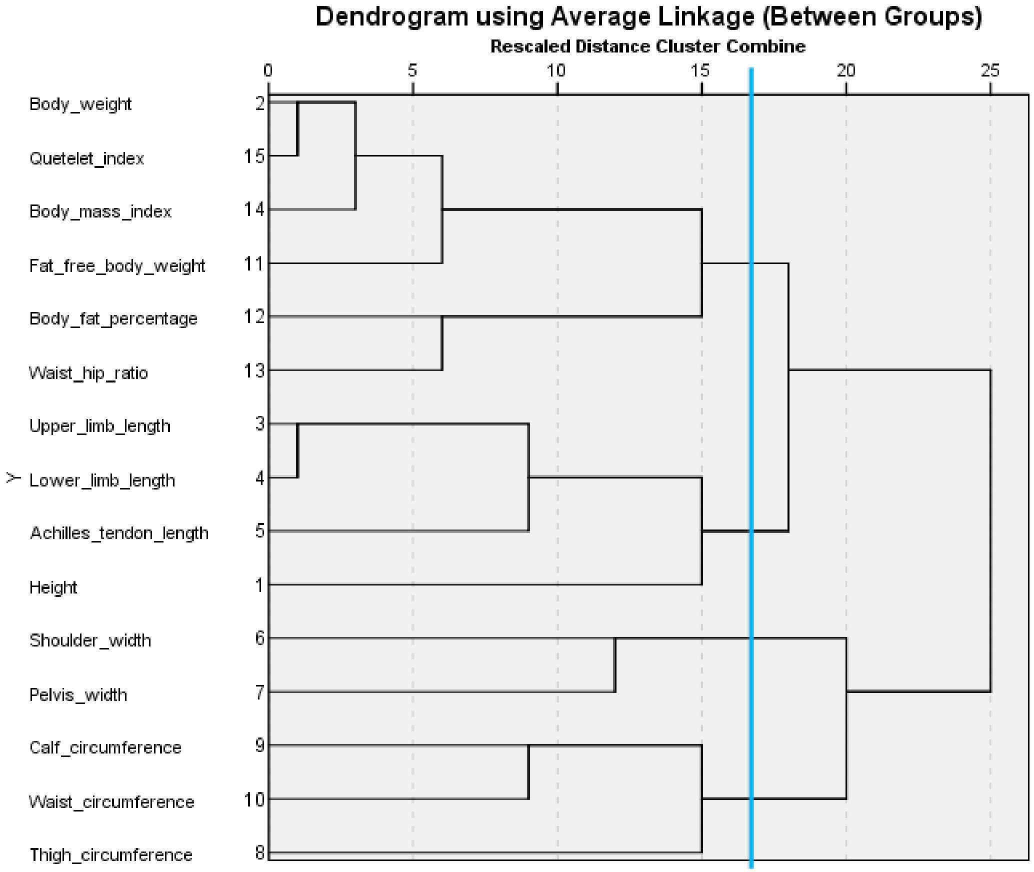

Figure 1 Dendrogram of cluster analysis for male athletes' body form

1.1.2 Regression analysis

Table 4 Representative body form indexes for male athletes in Freestyle Skiing Aerials (n=15)

**Indexes categorization**	**Indexes**						**Representative indexes**
Body length	Achilles tendon length	Lower limb length	Upper limb length	Height			Achilles tendon length
	0.913	0.842	0.912	0.772			
Body width	Shoulder width	Pelvis width					Pelvis width
	/	/					
Body circumference	Thigh circumference	Calf circumference	Waist circumference				Waist circumference
	0.830	0.672	0.884				
Body composition	Body weight	Fat-free body weight	Body fat percentage	Quetelet index	Waist-hip ratio	BMI	Quetelet index
	0.993	0.769	0.996	0.999	0.634	0.998	

Note: The numbers in the table represent the coefficients of determination, denoted as R-squared, from the multiple linear regression analysis

Table 5 Results of Multivariate linear regression analysis on the body length of male athletes

**Indexes (Dependent Variable/y)**	**Equation**	R^2	**P-Value of regression coefficient**
Achilles tendon length	y=22.731（±11.611）+0.356（±0.070）*Height-0.906（±0.178）*Upper limb length+0.023（±0.017）*Lower limb length	0.913	p＜0.0001
Lower limb length	y=-36.808（±216.281）-0.069（±2.062）*Height+0.300（±5.233）*Upper limb length+5.986（±4.494）*Achilles tendon length	0.842	0.0405（P＜0.05）
Upper limb length	y=28.799（±8.958）+0.346（±0.057）*Height+0.001（±0.017）*Lower limb length-0.776（±0.152）*Achilles tendon length	0.912	p＜0.0001
Height	y=-28.943（±30.437）+1.964（±0.388）*Achilles tendon length-0.001（±0.044）Lower limb length+2.226（±0.386）*Upper limb length	0.772	p＜0.0001

Table 6 Results of multivariate linear regression analysis on the body circumference of male athletes

Indexes (Dependent Variable/y)	Equation	R^2	P-Value of regression coefficient
Thigh circumference	y=-48.069（±32.445）+0.769（±0.504）*Waist circumference-0.749（±0.309）*Calf circumference	0.830	0.0120 (P＜0.05)
Calf circumference	y=-34.728（±25.088）＋1.296（±0.402）*Waist circumference-0.439（±0.181）*Thigh circumference	0.672	0.0197（P＜0.05）
Waist circumference	y=44.095（±6.291）＋0.358（±0.0.111）*Calf circumference＋0.286（±0.082）*Thigh circumference	0.884	0.0031(P＜0.01)

Table 7 Results of multivariate linear regression analysis on the body composition of male athletes

Indexes (Dependent Variable/y)	Equation	R^2	P-Value of regression coefficient
Body weight	y=4.259(±3.327)+0.366（±0.047）*Quetelet index-3.279(±0.464)*BMI-5.770(±3.923)*Waist-hip ratio-0.042（±0.116）*Body fat percentage-0.008（±0.146）*Fat-free body weight	0.993	p＜0.0001
Fat-free body weight	y=7.767(±7.816)-0.044（±0.759）*Body weight-3.055(±2.504)*BMI+4.993(±9.806)*Waist-hip ratio-0.763（±0.081）*Body fat percentage+0.330（±0.277）*Quetelet index	0.769	p＜0.0001
Body fat percentage	y=5.640(±10.110)-1.190（±0.127）*Fat-free body weight-0.336（±0.941）*Body weight+0.501（±0.333）*Quetelet index-4.604(±3.007)*BMI+9.774（±11.988）*Body weight	0.996	p＜0.0001
Quetelet index	y=-13.259 (±8.079)+0.412（±0.347）*Fat-free body weight+0.402（±0.267）*Body fat percentage+12.256（±10.346）*Waist-hip ratio+9.039(±0.246)*BMI+2.375（±0.306）*Body weight	0.999	p＜0.0001
Waist-hip ratio	y=0.657 (±0.168) + 0.006（±0.0118）*Fat-free body weight+0.007（±0.009）*Body fat percentage-0.102（±0.084）*BMI+0.011(±0.009) * Quetelet index-0.034（±0.023）*Body weight	0.634	0.0716（P＞0.05）
BMI	y=1.539 (±0.877)-0.046（±0.038）*Fat-free body weight-0.045（±0.029）*Body fat percentage-1.378（±1.137）*Waist-hip ratio+0.110(±0.003)*Quetelet index-0.258（±0.037）*Body weight	0.998	P＜0.001

1.2 Body form of female athletes

1.2.1 Cluster analysis

Table 8 Proximity matrix for clustering analysis of female athletes' body form

Proximity Matrix															
	Height	Body weight	Upper limb length	Lower limb length	Achilles tendon length	Shoulder width	Pelvis wide	Thigh circumference	Calf circumference	Waist circumference	Fat-free body weight	Body fat percentage	Waist-hip ratio	Body mass index	Quetelet index
Height	1.000	0.346	0.210	0.528	0.424	0.025	-0.109	0.173	0.083	0.243	-0.220	0.039	0.056	-0.104	0.174
Body weight	0.346	1.000	-0.050	0.000	-0.096	0.110	-0.024	0.072	-0.300	-0.050	0.108	0.746	0.791	0.811	0.960
Upper limb length	0.210	-0.050	1.000	0.216	0.506	0.149	0.040	0.192	-0.215	0.207	-0.617	0.143	0.110	-0.152	-0.154
Lower limb length	0.528	0.000	0.216	1.000	0.586	-0.145	-0.107	0.200	-0.161	-0.119	-0.036	-0.077	-0.211	-0.016	0.017
Achilles tendon length	0.424	-0.096	0.506	0.586	1.000	-0.135	-0.192	0.173	0.299	0.356	-0.307	-0.227	-0.296	-0.372	-0.226
Shoulder width	0.025	0.110	0.149	-0.145	-0.135	1.000	0.899	-0.312	0.021	-0.052	-0.081	-0.058	0.104	0.134	0.097
Pelvis wide	-0.109	-0.024	0.040	-0.107	-0.192	0.899	1.000	-0.606	-0.199	-0.341	-0.049	-0.006	0.086	0.087	0.006
Thigh circumference	0.173	0.072	0.192	0.200	0.173	-0.312	-0.606	1.000	0.218	0.548	0.293	-0.196	-0.129	0.023	0.070
Calf circumference	0.083	-0.300	-0.215	-0.161	0.299	0.021	-0.199	0.218	1.000	0.610	0.056	-0.667	-0.544	-0.510	-0.387
Waist circumference	0.243	-0.050	0.207	-0.119	0.356	-0.052	-0.341	0.548	0.610	1.000	-0.050	-0.471	-0.243	-0.391	-0.202
Fat-free body weight	-0.220	0.108	-0.617	-0.036	-0.307	-0.081	-0.049	0.293	0.056	-0.050	1.000	-0.055	0.094	0.316	0.280
Body fat percentage	0.039	0.746	0.143	-0.077	-0.227	-0.058	-0.006	-0.196	-0.667	-0.471	-0.055	1.000	0.912	0.798	0.786
Waist-hip ratio	0.056	0.791	0.110	-0.211	-0.296	0.104	0.086	-0.129	-0.544	-0.243	0.094	0.912	1.000	0.804	0.822
Body mass index	-0.104	0.811	-0.152	-0.016	-0.372	0.134	0.087	0.023	-0.510	-0.391	0.316	0.798	0.804	1.000	0.932
Quetelet index	0.174	0.960	-0.154	0.017	-0.226	0.097	0.006	0.070	-0.387	-0.202	0.280	0.786	0.822	0.932	1.000

Table 9 Agglomeration schedule for clustering analysis of female athletes' body form

Agglomeration Schedule						
Stage	Cluster Combined		Coefficients	Stage Cluster First Appears		Next Stage
	Cluster 1	Cluster 2		Cluster 1	Cluster 2	
1	2	15	.960	0	0	4
2	12	13	.912	0	0	5
3	6	7	.899	0	0	13
4	2	14	.872	1	0	5
5	2	12	.791	4	2	11
6	9	10	.610	0	0	9
7	4	5	.586	0	0	8
8	1	4	.476	0	7	10
9	8	9	.383	0	6	12
10	1	3	.310	8	0	12
11	2	11	.148	5	0	13
12	1	8	.119	10	9	14
13	2	6	.034	11	3	14
14	1	2	-.140	12	13	0

Table 10 Cluster membership of female athletes' body form

**Cluster Membership**	
Case	4 Clusters
Height	1
Body weight	2
Upper limb length	1
Lower limb length	1
Achilles tendon length	1
Shoulder width	3
Pelvis wide	3
Thigh circumference	4
Calf circumference	4
Waist circumference	4
Fat-free body weight	2
Body fat percentage	2
Waist-hip ratio	2
Body mass index	2
Quetelet index	2

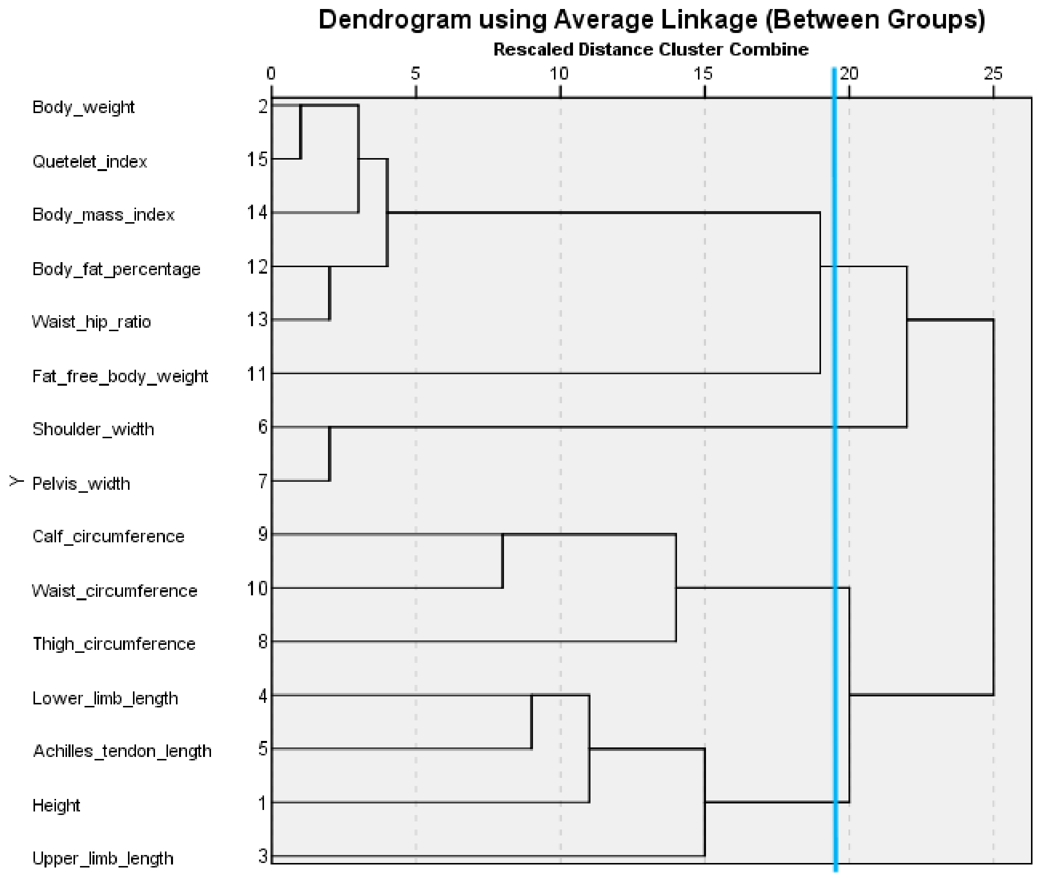

Figure 2 Dendrogram of cluster analysis for female athletes' body form

1.2.2 Regression analysis

Table 11 Representative body form indexes for female athletes in Freestyle Skiing Aerials（n=14）

**Indexes categorization**	**Indexes**						**Representative indexes**
Body length	Achilles tendon length	Lower limb length	Upper limb length	Height			Achilles tendon length
	0.940	0.811	0.802	0.836			
Body width	Shoulder width	Pelvis width					Pelvis width
	/	/					
Body circumference	Thigh circumference	Calf circumference	Waist circumference				Waist circumference
	0.818	0.684	0.920				
Body composition	Body weight	Fat-free body weight	Body fat percentage	Quetelet index	Waist-hip ratio	BMI	Quetelet index
	0.990	0.985	0.992	0.997	0.883	0.983	

Note: The numbers in the table represent the coefficients of determination, denoted as R-squared, from the multiple linear regression analysis

Table 12 Results of Multivariate linear regression analysis on the body length of female athletes

**Indexes (Dependent Variable/y)**	**Equation**	**R^2**	**P-Value of regression coefficient**
Achilles tendon length	y=-12.469（±6.515）+0.162（±0.034）*Height-0.036（±0.014）*Upper limb length+0.098（±0.027）*Lower limb length	0.940	0.0039（P＜0.01）
Lower limb length	y=144.44（±36.638）-1.115（±0.304）*Height+0.255（±0.110）*Upper limb length+5.789（±1.600）*Achilles tendon length	0.811	0.0149（P＜0.05）
Upper limb length	y=-260.431（±108.395）+2.673（±0.711）*Height+1.379（±0.592）*Lower limb length-11.470（±4.355）*Achilles tendon length	0.802	0.0266（P＜0.05）
Height	y=107.424（±19.121）+4.254（±0.900）*Achilles tendon length-0.512（±0.135）Lower limb length+0.219（±0.058）*Upper limb length	0.836	0.0006（P＜0.001）

Table 13 Results of multivariate linear regression analysis on the body circumference of female athletes

Indexes (Dependent Variable/y)	Equation	R^2	P-Value of regression coefficient
Thigh circumference	y=-9.977（±23.121）+1.123（±0.345）*Waist circumference-0.449（±0.656）*Calf circumference	0.818	0.0180（P＜0.05）
Calf circumference	y=14.717（±9.517）-0.091（±0.133）*Thigh circumference+0.356（±0.189）*Waist circumference	0.684	0.1594（P＞0.05）
Waist circumference	y=22.562（±12.856）＋0.683（±0.363）*Calf circumference＋0.437（±0.134）*Thigh circumference	0.920	P＜0.001

Table 14 Results of multivariate linear regression analysis on the body composition of female athletes

Indexes (Dependent Variable/y)	Equation	R^2	P-Value of regression coefficient
Body weight	y=--16.685 (±7.938)+1.771（±0.560）*Fat-free body weight+1.146（±0.349）*Body fat percentage-13.754（±12.593）*Waist-hip ratio+0.625(±0.945)*BMI-0.091（±0.128）*Quetelet index	0.990	P＜0.001
Fat-free body weight	y=7.511 (±3.204)+8.355（±4.852）*Waist-hip ratio-0.900(±0.256)*BMI+0.129（±0.032）*Quetelet index+0.314（±0.099）*Body weight-0.629（±0.033）*Body fat percentage	0.985	P＜0.001
Body fat percentage	y=-10.048 (±5.490)-1.554（±0.082）*Fat-free body weight+15.262（±7.109）*Waist-hip ratio-1.348(±0.430)*BMI+0.196（±0.054）*Quetelet index+0.501（±0.153）*Body weight	0.992	P＜0.001
Quetelet index	y=-32.809 (±23.746)+5.201（±1.288）*Fat-free body weight+3.196（±0.875）*Body fat percentage-41.576（±34.942）*Waist-hip ratio+7.135(±0.603)*BMI-0.649（±0.916）*Body weight	0.997	P＜0.001
Waist-hip ratio	y=0.177（±0.251）+0.029（±0.023）*BMI+0.032（±0.019）*Fat-free body weight+0.024（±0.003）*Quetelet index-0.009（±0.009）*Body weight	0.883	0.0008（P＜0.001）
BMI	y=4.580（±3.218）-0.674(±0.192)*Fat-free body weight-0.409（±0.130）*Body fat percentage0.133（±0.011）*Quetelet index+0.083（±0.125）*Body weight+5.691（±4.486）*Waist-hip ratio	0.983	P＜0.001

2 Physiological function

2.1 Physiological function of male athletes

2.1.1 Cluster analysis

Table 15 Proximity matrix for clustering analysis of male athletes' physiological function

Proximity Matrix									
	Maximum anaerobic power	Relative maximum anaerobic power	Maximal oxygen intake	Relative maximum oxygen uptake	Hemoglobin	Red blood cell count	Serum testosterone	Blood urea	Serum cortisol
Maximum anaerobic power	1.000	0.808	0.434	0.088	-0.484	-0.653	-0.203	-0.098	-0.365
Relative maximum anaerobic power	0.808	1.000	0.312	-0.045	-0.361	-0.416	-0.317	-0.222	-0.410
Maximal oxygen intake	0.434	0.312	1.000	0.476	-0.317	-0.376	-0.258	-0.406	-0.493
Relative maximum oxygen uptake	0.088	-0.045	0.476	1.000	0.246	0.213	0.025	0.246	0.187
Hemoglobin	-0.484	-0.361	-0.317	0.246	1.000	0.934	0.459	0.283	0.339
Red blood cell count	-0.653	-0.416	-0.376	0.213	0.934	1.000	0.430	0.241	0.357
Serum testosterone	-0.203	-0.317	-0.258	0.025	0.459	0.430	1.000	0.455	0.371
Blood urea	-0.098	-0.222	-0.406	0.246	0.283	0.241	0.455	1.000	0.923
Serum cortisol	-0.365	-0.410	-0.493	0.187	0.339	0.357	0.371	0.923	1.000

Table 16 Agglomeration schedule for clustering analysis of male athletes' physiological function

Agglomeration Schedule						
Stage	Cluster Combined		Coefficients	Stage Cluster First Appears		Next Stage
	Cluster 1	Cluster 2		Cluster 1	Cluster 2	
1	5	6	0.934	0	0	5
2	8	9	0.923	0	0	6
3	1	2	0.808	0	0	7
4	3	4	0.476	0	0	7
5	5	7	0.445	1	0	6
6	5	8	0.341	5	2	8
7	1	3	0.197	3	4	8
8	1	5	-0.223	7	6	0

Table 17 Cluster membership of male athletes' physiological function

Cluster Membership	
Case	3 Clusters
Maximum anaerobic power	1
Relative maximum anaerobic power	1
Maximal oxygen intake	2
Relative maximum oxygen uptake	2
Hemoglobin	3
Red blood cell count	3
Serum testosterone	3
Blood urea	3
Serum cortisol	3

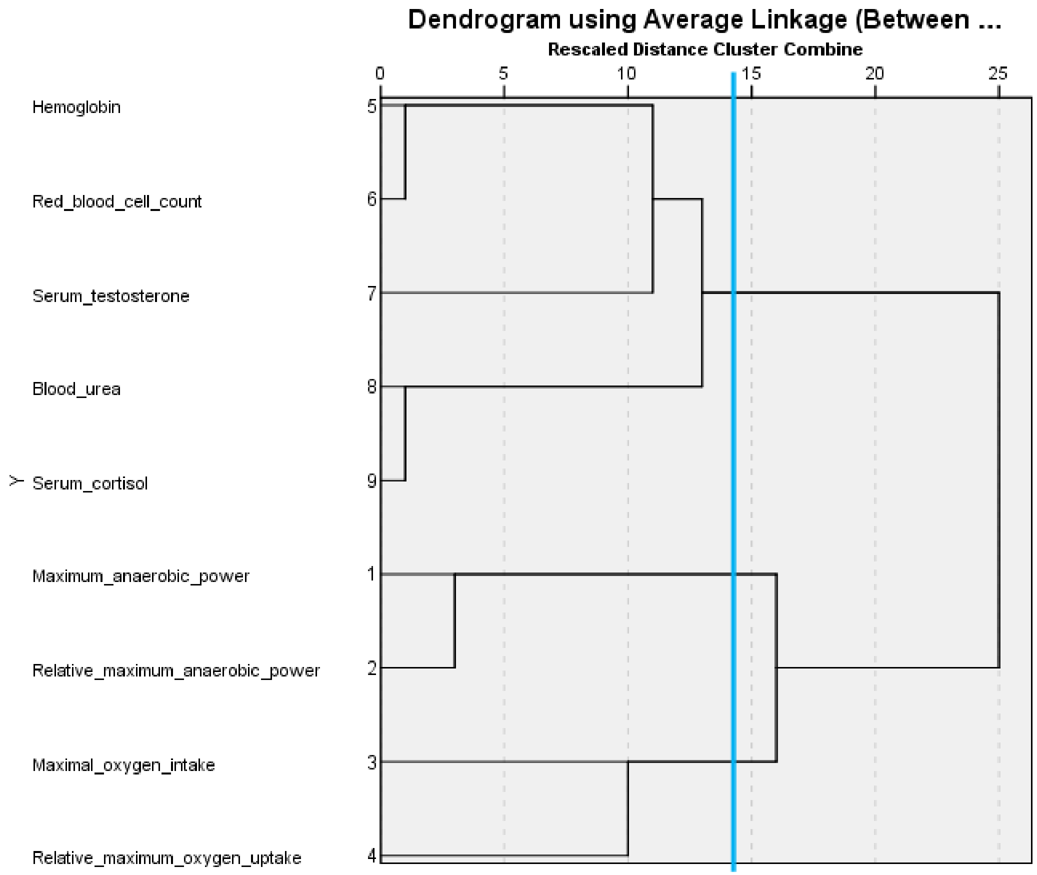

Figure 3 Dendrogram of cluster analysis for male athletes' physiological function

2.1.2 Regression analysis

Table 18 Representative physiological function indexes for male athletes in Freestyle Skiing Aerials（n=15）

**Indexes categorization**	**Indexes**					**Representative indexes**
Anaerobic capacity	Maximum anaerobic power	Relative maximum anaerobic power				Relative maximum anaerobic power
	/	/				
Cardiopulmonary performance	Maximal oxygen intake	Relative maximum oxygen uptake				Relative maximum oxygen uptake
	/	/				
Exercise biochemical ability	Hemoglobin	Red-cell count	Blood urea	Serum testosterone	Serum cortisol	Hemoglobin
	0.910	0.906	0.424	0.471	0.459	

Note: The numbers in the table represent the coefficients of determination, denoted as R-squared, from the multiple linear regression analysis

Table 19 Results of Multivariate linear regression analysis on the exercise biochemical ability of male athletes

Indexes (Dependent Variable/y)	Equation	R^2	P-Value of regression coefficient
Hemoglobin	y=11.273（±16.394）+24.762（±2.854）*Red-cell count-0.005（±0.006）*Serum testosterone+1.445（±0.845）*Blood urea+0.427（±0.765）*Serum cortisol	0.910	P＜0.001
Red-cell count	y=-0.047（±0.636）+0.036（±0.004）*Hemoglobin-0.00022（±0.00023）*Serum testosterone-0.044（±0.034）*Blood ureat-0.0014(±0.029)*Serum cortisol	0.906	P＜0.001
Blood urea	y=5.012（±5.289）-3.334（±2.532）*Red-cell count+0.156（±0.092）*Hemoglobin+0.00047（±0.00211）*Serum testosterone-0.421（±0.218）*Serum cortisol	0.424	0.1972（P＞0.05）
Serum testosterone	y=1065.069（±754..697）-374.155（±393.140）*Red-cell count-11.181（±15.171）*Hemoglobin-10.629（±47.212）*Blood urea-44.199（±35.647）*Serum cortisol	0.471	0.6012（P＞0.05）
Serum cortisol	y=8.997（±6.204）-0.164（±3.390）*Red-cell count+0.071（±0.127）*Hemoglobin-0.644（±0.344）*Blood urea-0.003（±0.002）*Serum testosterone	0.459	0.1532（P＞0.05）

2.2 Physiological function of female athletes

2.2.1 Cluster analysis

Table 20 Proximity matrix for clustering analysis of female athletes' physiological function

Proximity Matrix									
	Maximum anaerobic power	Relative maximum anaerobic power	Maximal oxygen intake	Relative maximum oxygen uptake	Hemoglobin	Red blood cell count	Serum testosterone	Blood urea	Serum cortisol
Maximum anaerobic power	1.000	0.919	-0.234	0.294	-0.370	-0.248	0.058	-0.050	0.295
Relative maximum anaerobic power	0.919	1.000	-0.193	0.365	-0.287	-0.160	0.093	-0.145	0.242
Maximal oxygen intake	-0.234	-0.193	1.000	0.487	0.286	0.281	-0.231	-0.190	-0.145
Relative maximum oxygen uptake	0.294	0.365	0.487	1.000	0.019	0.027	-0.192	-0.198	0.201
Hemoglobin	-0.370	-0.287	0.286	0.019	1.000	0.357	0.180	0.333	0.338
Red blood cell count	-0.248	-0.160	0.281	0.027	0.357	1.000	0.041	0.069	0.054
Serum testosterone	0.058	0.093	-0.231	-0.192	0.180	0.041	1.000	0.369	0.446
Blood urea	-0.050	-0.145	-0.190	-0.198	0.333	0.069	0.369	1.000	0.828
Serum cortisol	0.295	0.242	-0.145	0.201	0.338	0.054	0.446	0.828	1.000

Table 21 Agglomeration schedule for clustering analysis of female athletes' physiological function

Agglomeration Schedule						
Stage	Cluster Combined		Coefficients	Stage Cluster First Appears		Next Stage
	Cluster 1	Cluster 2		Cluster 1	Cluster 2	
1	1	2	0.919	0	0	7
2	8	9	0.828	0	0	4
3	3	4	0.487	0	0	7
4	7	8	0.408	0	2	6
5	5	6	0.357	0	0	6
6	5	7	0.169	5	4	8
7	1	3	0.058	1	3	8
8	1	5	-0.036	7	6	0

Table 22 Cluster membership of female athletes' physiological function

Cluster Membership	
Case	3 Clusters
Maximum anaerobic power	1
Relative maximum anaerobic power	1
Maximal oxygen intake	2
Relative maximum oxygen uptake	2
Hemoglobin	3
Red blood cell count	3
Serum testosterone	3
Blood urea	3
Serum cortisol	3

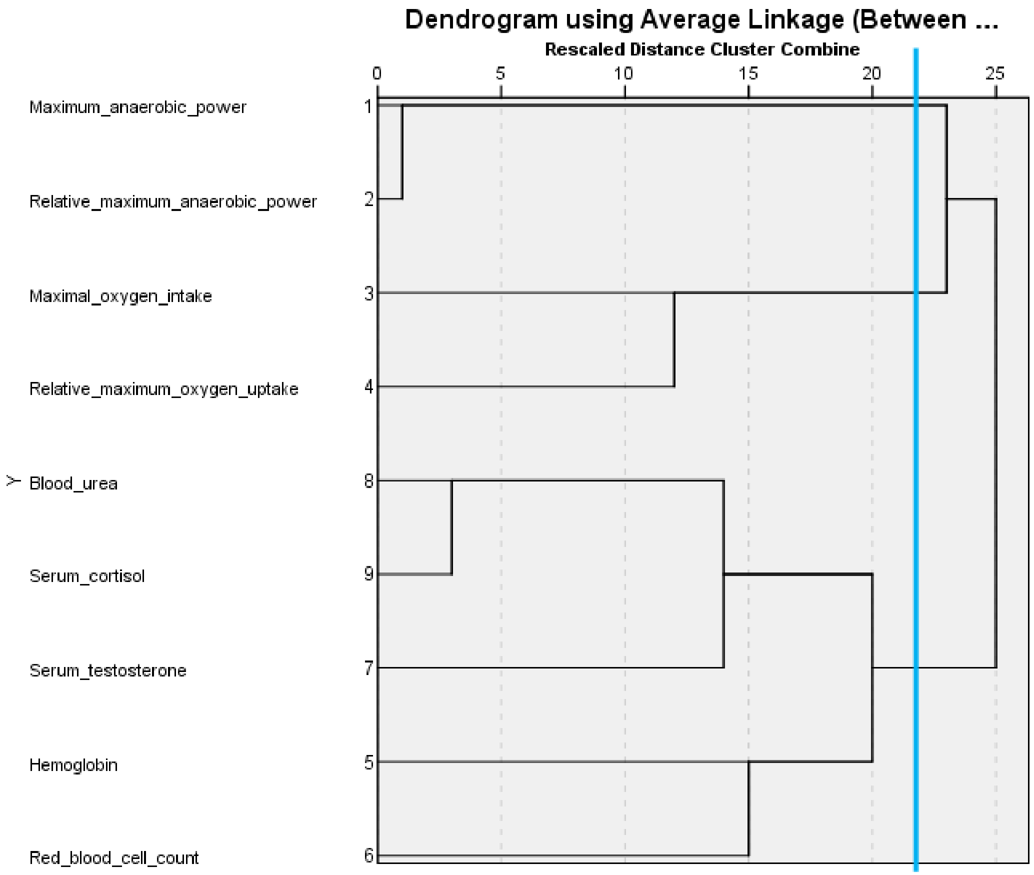

Figure 4 Dendrogram of cluster analysis for female athletes' physiological function

2.2.2 Regression analysis

Table 23 Representative physiological function indexes for female athletes in Freestyle Skiing Aerials（n=14）

Indexes categorization	Indexes					Representative indexes
Anaerobic capacity	Maximum anaerobic power	Relative maximum anaerobic power				Relative maximum anaerobic power
	/	/				
Cardiopulmonary performance	Maximal oxygen intake	Relative maximum oxygen uptake				Relative maximum oxygen uptake
	/	/				
Exercise biochemical ability	Hemoglobin	Red-cell count	Blood urea	Serum testosterone	Serum cortisol	Hemoglobin
	0.672	0.414	0.583	0.670	0.487	

Note: The numbers in the table represent the coefficients of determination, denoted as R-squared, from the multiple linear regression analysis

Table 24 Results of Multivariate linear regression analysis on the exercise biochemical ability of female athletes

Indexes (Dependent Variable/y)	Equation	R^2	P-Value of regression coefficient
Hemoglobin	y=57.486（±20.425）+8.273（±2.399）*Red-cell count+0.309（±0.075）*Serum testosterone-2.506（±1.605）*Blood urea+1.954（±0.678）*Serum cortisol	0.672	0.0071（P＜0.01）
Red-cell count	y=-1.017（±2.531）+0.069（±0.020）*Hemoglobin-0.025（±0.008）*Serum testosterone+0.138（±0.158）*Blood ureat-0.182(±0.060)*Serum cortisol	0.414	0.0246（P＜0.05）
Blood urea	y=9.595（±4.049）+0.563（±0.647）*Red-cell count-0.085（±0.054）*Hemoglobin+0.037（±0.020）*Serum testosterone+0.087（±0.171）*Serum cortisol	0.583	0.3891（P＞0.05）
Serum testosterone	y=-99.266（±65.286）-20.679（±6.613）*Red-cell count+2.112（±0.513）*Hemoglobin+7.492（±4.015）*Blood urea-4.134（±2.033）*Serum cortisol	0.670	0.0133（P＜0.05）
Serum cortisol	y=-1.995（±9.911）-2.759（±0.914）*Red-cell count+0.246（±0.085）*Hemoglobin+0.321（±0.633）*Blood urea-0.076（±0.037）*Serum testosterone	0.487	0.0715（P＞0.05）

3 Physical capacity

3.1 Physical capacity of male athletes

3.1.1 Cluster analysis

Table 25 Proximity matrix for clustering analysis of male athletes' physical capacity

Proximity Matrix														
	Squat up	Pull up	Back throw	Side throw (left)	Side throw (right)	Squat on the balance pad with the barbell raising	Quick v-up	Single-leg triple jump (left)	Single-leg triple jump (right)	Standing long jump	Power clean	30-meter sprint	12-minute run	Agile running
Squat up	1.000	0.698	-0.133	-0.422	-0.633	-0.133	-0.044	0.330	0.410	0.234	0.623	-0.401	0.211	-0.651
Pull up	0.698	1.000	0.029	-0.287	-0.427	-0.022	0.150	0.382	0.467	0.564	0.358	-0.642	0.418	-0.790
Back throw	-0.133	0.029	1.000	0.869	0.773	0.320	0.335	-0.420	-0.328	-0.204	-0.686	0.002	0.212	-0.016
Side throw (left)	-0.422	-0.287	0.869	1.000	0.912	0.190	0.217	-0.600	-0.525	-0.398	-0.815	0.241	0.069	0.302
Side throw (right)	-0.633	-0.427	0.773	0.912	1.000	0.417	0.369	-0.656	-0.656	-0.418	-0.881	0.296	-0.084	0.520
Squat on the balance pad with the barbell raising	-0.133	-0.022	0.320	0.190	0.417	1.000	0.878	-0.032	-0.124	-0.111	-0.328	-0.024	-0.456	0.247
Quick v-up	-0.044	0.150	0.335	0.217	0.369	0.878	1.000	0.055	-0.064	-0.108	-0.223	-0.051	-0.421	0.069
Single-leg triple jump (left)	0.330	0.382	-0.420	-0.600	-0.656	-0.032	0.055	1.000	0.807	0.529	0.467	-0.315	0.062	-0.609
Single-leg triple jump (right)	0.410	0.467	-0.328	-0.525	-0.656	-0.124	-0.064	0.807	1.000	0.733	0.633	-0.638	0.216	-0.647
Standing long jump	0.234	0.564	-0.204	-0.398	-0.418	-0.111	-0.108	0.529	0.733	1.000	0.410	-0.838	0.545	-0.577
Power clean	0.623	0.358	-0.686	-0.815	-0.881	-0.328	-0.223	0.467	0.633	0.410	1.000	-0.381	0.083	-0.394
30-meter sprint	-0.401	-0.642	0.002	0.241	0.296	-0.024	-0.051	-0.315	-0.638	-0.838	-0.381	1.000	-0.348	0.594

12-minute run	0.211	0.418	0.212	0.069	-0.084	-0.456	-0.421	0.062	0.216	0.545	0.083	-0.348	1.000	-0.475
Agile running	-0.651	-0.790	-0.016	0.302	0.520	0.247	0.069	-0.609	-0.647	-0.577	-0.394	0.594	-0.475	1.000

Table 26 Agglomeration schedule for clustering analysis of male athletes' physical capacity

**Agglomeration Schedule**						
Stage	Cluster Combined		Coefficients	Stage Cluster First Appears		Next Stage
	Cluster 1	Cluster 2		Cluster 1	Cluster 2	
1	4	5	0.912	0	0	3
2	6	7	0.878	0	0	10
3	3	4	0.821	0	1	10
4	8	9	0.807	0	0	6
5	1	2	0.698	0	0	9
6	8	10	0.631	4	0	8
7	12	14	0.594	0	0	12
8	8	11	0.503	6	0	9
9	1	8	0.421	5	8	11
10	3	6	0.308	3	2	12
11	1	13	0.256	9	0	13
12	3	12	0.158	10	7	13
13	1	3	-0.364	11	12	0

Table 27 Cluster membership of male athletes' physical capacity

**Cluster Membership**	
Case	4 Clusters
Squat up	1
Pull up	1
Back throw	2
Side throw (left)	2
Side throw (right)	2
Squat on the balance pad with the barbell raising	2
Quick v-up	2
Single-leg triple jump (left)	1
Single-leg triple jump (right)	1
Standing long jump	1
Power clean	1
30-meter sprint	3
12-minute run	4
Agile running	3

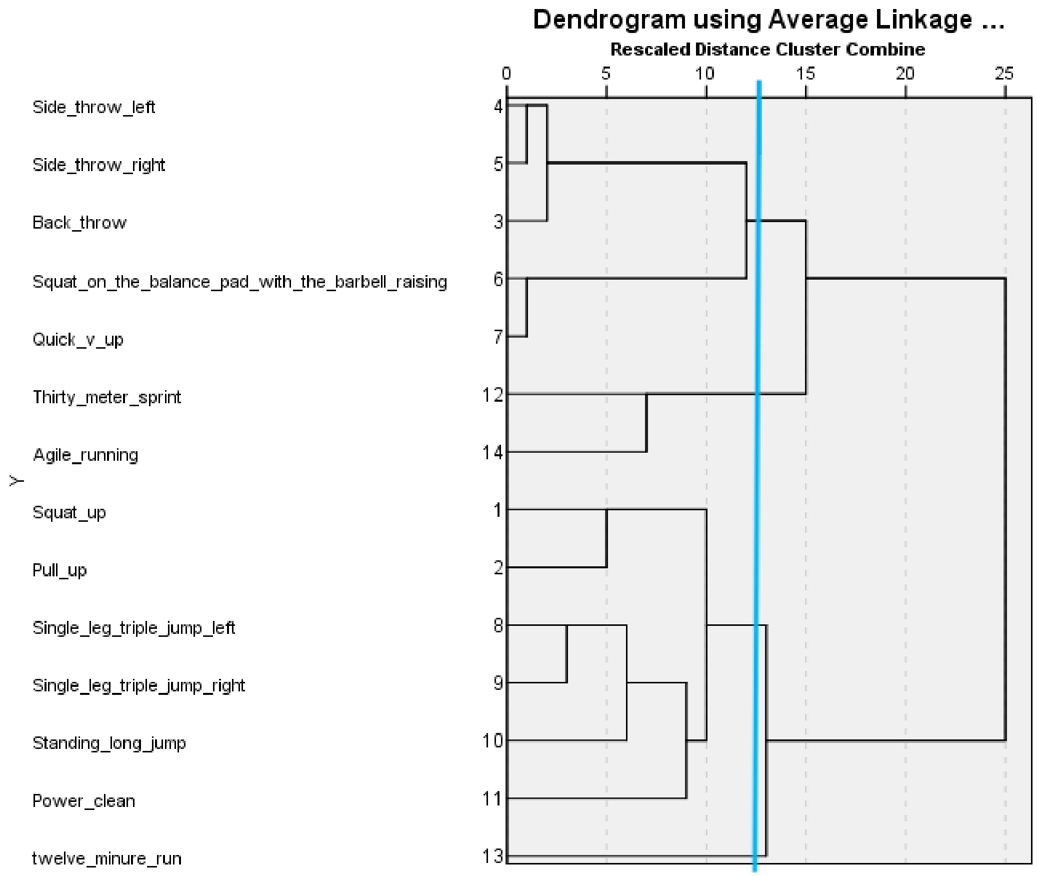

Figure 5 Dendrogram of cluster analysis for male athlete physical capacity

3.1.2 Regression analysis

Table 28 Representative physical quality indexes for male athletes in Freestyle Skiing Aerials（n=15）

**Indexes Categorization**	**Indexes**						**Representative indexes**
Limb strength	Squat up	Pull up	Power clean	Standing long jump	Single-leg triple jump (left)	Single-leg triple jump (right)	Power clean
	0.747	0.726	0.827	0.709	0.667	0.730	
Core strength	Side throw (right)	Side throw（left）	Back throw	Squat on the balance pad with the barbell raising	Quick v-up		Squat on the balance pad with the barbell raising
	0.587	0.669	0.618	0.814	0.603		
Speed-agility	Agile running	30-meter sprint					30-meter sprint
	/	/					
Aerobic capacity	12-minute run						12-minute run
	/						

Note: The numbers in the table represent the coefficients of determination, denoted as R-squared, from the multiple linear regression analysis

Table 29 Results of Multivariate linear regression analysis on the limb strength of male athletes

Indexes (Dependent Variable/y)	Equation	R^2	P-Value of regression coefficient
Squat up	y=188.164（±55.388）+2.177（±0.613）*Pull up-0.941（±8.518）*Single-leg triple jump (left) +3.475（±12.672）*Single-leg triple jump (right)-0.472（±0.290）*Standing long jump+0.358(±0.163)*Power clean	0.747	0.0151（P＜0.05）
Pull up	y=-65.744（±19.533）+0.782（±2.979）*Single-leg triple jump (left)-1.444(±4.438)*Single-leg triple jump (right)+0.204（±0.094）*Standing long jump-0.079（0.066）*Power clean+0.268（±0.075）*Squat up	0.726	0.0104（P＜0.05）
Power clean	y=-215.925（±117.685）-3.368（±14.008）*Single-leg triple jump (left)+20.174（±19.872）*Single-leg triple jump (right)+0.266（±0.537）*Standing long jump+0.974（±0.443）*Squat up-1.753（±1.453）*Pull up	0.827	0.0208（P＜0.05）
Standing long jump	y=187.634（±56.833）-4.729（±8.463）*Single-leg triple jump (left)+20.842(±10.812)*Single-leg triple jump (right)+0.100（±0.201）*Power clean-0.482（0.296）*Squat up+1.687（±0.778）*Pull up	0.709	0.0267（P＜0.05）
Single-leg triple jump (left)	y=1.806(±3.216)+1.035(±0.359)*Single-leg triple jump (right)-0.001(±0.13)*Squat up+0.010（±0.037）*Pull up-0.007（±0.013）*Standing long jump-0.002（±0.008）*Power clean	0.667	0.0455（P＜0.05）
Single-leg triple jump (right)	y=-0.615（±2.182）+0.002（±0.009）*Squat up-0.008(±0.025)*Pull up+0.014（±0.007）*Standing long jump+0.005（±0.005）*Power clean+0.465（±0.161）*Single-leg triple jump (left)	0.730	0.0028（P＜0.01）

Table 30 Results of Multivariate linear regression analysis on the core strength of male athletes

Indexes (Dependent Variable/y)	Equation	R^2	P-Value of regression coefficient
Side throw (right)	y=7.023（±2.960）+0.010（±0.205）*Side throw （left）-0.173（±0.229）*Back throw+0.320（±0.307）*Quick v-up-0.198（±0.093）*Squat on the balance pad with the barbell raising	0.587	0.1218（P＞0.05）
Side throw（left）	y=10.400（±4.600）+0.025（±0.487）*Side throw (right)+0.068（±0.362）*Back throw-0.206（±0.494）*Quick v-up-0.234（±0.156）*Squat on the balance pad with the barbell raising	0.669	0.0571（P＞0.05）
Back throw	y=1.481（±4.952）+0.052（±0.275）*Side throw（left）-0.312（±0.413）*Side throw (right)+0.959（±0.311）*Quick v-up-0.292（±0.119）*Squat on the balance pad with the barbell raising	0.618	0.0334（P＜0.05）
Squat on the balance pad with the barbell raising	y=17.920 (±8.759)-0.782（±0.523）*Side throw（left）-1.574（±0.740）*Side throw (right)-1.289（±0.524）*Back throw+1.699(±0.753)*Quick v-up	0.814	0.0011（P＜0.001）
Quick v-up	y=1.481（±4.952）+0.052（±0.275）*Side throw（left）-0.312（±0.413）*Side throw (right)+0.959（±0.311）*Quick v-up-0.292（±0.119）*Squat on the balance pad with the barbell raising	0.603	0.0394（P＜0.05）

3.2 Physical capacity of female athletes

3.2.1 Cluster analysis

Table 31 Proximity matrix for clustering analysis of female athletes' physical capacity

Proximity Matrix														
	Squat up	Pull up	Back throw	Side throw (left)	Side throw (right)	Squat on the balance pad with the barbell raising	Quick v-up	Single-leg triple jump (left)	Single-leg triple jump (right)	Standing long jump	Power clean	30-meter sprint	Agile running	12-minute run
Squat up	1.000	0.226	0.184	0.562	0.413	0.626	-0.030	0.445	0.534	0.842	0.829	-0.281	-0.677	-0.021
Pull up	0.226	1.000	0.111	0.356	0.498	0.125	0.107	0.369	0.446	0.456	0.328	-0.444	-0.304	-0.441
Back throw	0.184	0.111	1.000	0.362	0.415	0.345	-0.033	0.215	0.055	-0.061	0.208	-0.251	-0.191	-0.552
Side throw (left)	0.562	0.356	0.362	1.000	0.833	0.736	0.498	0.027	0.208	0.423	0.597	-0.312	-0.453	-0.075
Side throw (right)	0.413	0.498	0.415	0.833	1.000	0.687	0.457	0.075	0.094	0.312	0.462	-0.514	-0.474	-0.435
Squat on the balance pad with the barbell raising	0.626	0.125	0.345	0.736	0.687	1.000	0.340	0.097	0.238	0.419	0.660	-0.228	-0.612	-0.250
Quick v-up	-0.030	0.107	-0.033	0.498	0.457	0.340	1.000	-0.424	-0.150	0.080	-0.253	-0.013	0.072	0.176
Single-leg triple jump (left)	0.445	0.369	0.215	0.027	0.075	0.097	-0.424	1.000	0.738	0.493	0.385	-0.340	-0.463	-0.286
Single-leg triple jump (right)	0.534	0.446	0.055	0.208	0.094	0.238	-0.150	0.738	1.000	0.704	0.524	-0.181	-0.431	-0.150
Standing long jump	0.842	0.456	-0.061	0.423	0.312	0.419	0.080	0.493	0.704	1.000	0.682	-0.147	-0.506	-0.105

Power clean	0.829	0.328	0.208	0.597	0.462	0.660	-0.253	0.385	0.524	0.682	1.000	-0.251	-0.667	-0.235
30-meter sprint	-0.281	-0.444	-0.251	-0.312	-0.514	-0.228	-0.013	-0.340	-0.181	-0.147	-0.251	1.000	0.639	0.216
Agile running	-0.677	-0.304	-0.191	-0.453	-0.474	-0.612	0.072	-0.463	-0.431	-0.506	-0.667	0.639	1.000	0.263
12-minute run	-0.021	-0.441	-0.552	-0.075	-0.435	-0.250	0.176	-0.286	-0.150	-0.105	-0.235	0.216	0.263	1.000

Table 32 Agglomeration schedule for clustering analysis of female athletes' physical capacity

Agglomeration Schedule						
Stage	Cluster Combined		Coefficients	Stage Cluster First Appears		Next Stage
	Cluster 1	Cluster 2		Cluster 1	Cluster 2	
1	1	10	0.842	0	0	3
2	4	5	0.833	0	0	5
3	1	11	0.755	1	0	7
4	8	9	0.738	0	0	7
5	4	6	0.712	2	0	8
6	12	13	0.639	0	0	11
7	1	8	0.514	3	4	9
8	4	7	0.432	5	0	10
9	1	2	0.365	7	0	12
10	3	4	0.272	0	8	12
11	12	14	0.239	6	0	13
12	1	3	0.208	9	10	13
13	1	12	-0.304	12	11	0

Table 33 Cluster membership of female athletes’ physical capacity

**Cluster Membership**	
Case	4 Clusters
Squat up	1
Pull up	1
Back throw	2
Side throw (left)	2
Side throw (right)	2
Squat on the balance pad with the barbell raising	2
Quick v-up	2
Single-leg triple jump (left)	1
Single-leg triple jump (right)	1
Standing long jump	1
Power clean	1
30-meter sprint	3
Agile running	3
12-minute run	4

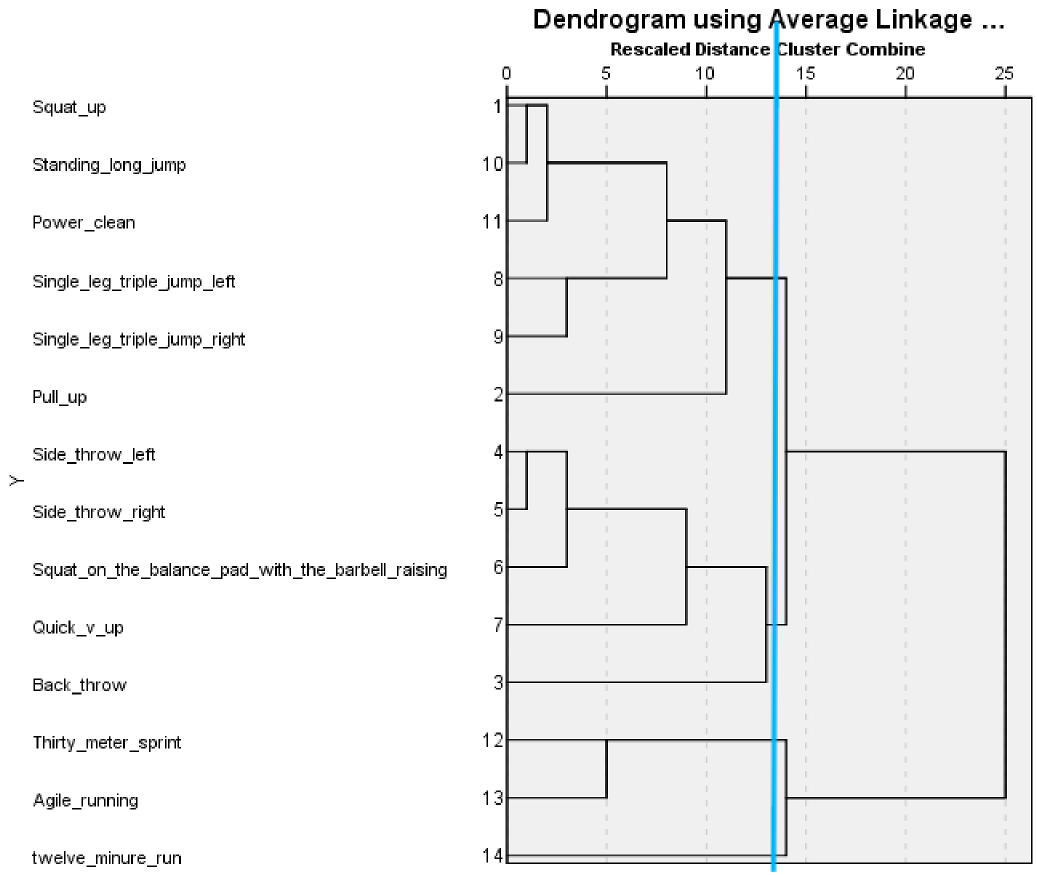

Figure 6 Dendrogram of cluster analysis for female athletes' physical capacity

3.2.2 Regression analysis

Table 34 Representative physical quality indexes for female athletes in Freestyle Skiing Aerials（n=14）

**Indexes Categorization**	**Indexes**						**Representative indexes**
Limb strength	Squat up	Pull up	Power clean	Standing long jump	Single-leg triple jump (left)	Single-leg triple jump (right)	Power clean
	0.745	0.503	0.884	0.855	0.695	0.740	
core strength	Side throw (right)	Side throw（left）	Back throw	Squat on the balance pad with the barbell raising	Quick v-up		Squat on the balance pad with the barbell raising
	0.674	0.724	0.579	0.845	0.627		
speed sensitivity	Agile running	30-meter sprint					30-meter sprint
	/	/					
aerobic capacity	12-minute run						12-minute run
	/						

Note: The numbers in the table represent the coefficients of determination, denoted as R-squared, from the multiple linear regression analysis

Table 35 Results of Multivariate linear regression analysis on the limb strength of female athletes

Indexes (Dependent Variable/y)	Equation	R^2	P-Value of regression coefficient
Squat up	y=-99.177（±37.314）-0.884（±0.605）*Pull up+4.043（±4.223）*Single-leg triple jump (left) -4.167（±3.806）*Single-leg triple jump (right)+0.754（±0.222）*Standing long jump+0.646(±0.220）*Power clean	0.745	0.0014（P＜0.05）
Pull up	y=--34.456（±23.625）+1.407（±2.261）*Single-leg triple jump (left)-0.471(±2.112)*Single-leg triple jump (right)+0.235（±0.160）*Standing long jump+0.156（±0.155）*Power clean-0.238（±0.163）*Squat up	0.503	0.4378（P＞0.05）
Power clean	y=45.363（±54.762）-2.967（±4.857）*Single-leg triple jump (left)+3.719（±4.354）**Single-leg triple jump (right)-0.359（±0.365）*Standing long jump+0.803（±0.273）*Squat up+0.719（±0.716）*Pull up	0.884	0.0208（P＜0.05）
Standing long jump	y=140.196（±16.266）-3.768（±4.343）*Single-leg triple jump (left)+6.345(±3.501)*Single-leg triple jump (right)-0.300（±0.305）*Power clean+0.783（±0.231）*Squat up+0.907（±0.615）*Pull up	0.855	0.0033（P＜0.01）
Single-leg triple jump (left)	y=5.352(±3.592)+0.604(±0.243)*Single-leg triple jump (right)+0.025(±0.027)*Squat up+0.033（±0.053）*Pull up-0.023（±0.026）*Standing long jump-0.015（±0.025）*Power clean	0.695	0.1349（P＞0.05）
Single-leg triple jump (right)	y=-6.643（±3.763）-0.031（±0.029）*Squat up-0.013(±0.059)*Pull up+0.046（±0.025）*Standing long jump+0.022（±0.026）*Power clean+0.721（±0.290）*Single-leg triple jump (left)	0.740	0.0290（P＜0.05）

Table 36 Results of Multivariate linear regression analysis on the core strength of female athletes

Indexes (Dependent Variable/y)	Equation	R^2	P-Value of regression coefficient
Side throw (right)	y=1.879（±2.411）+0.658（±0.280）*Side throw （left）+0.114（±0.447）*Back throw-0.033（±0.129）*Quick v-up-0.034（±0.051）*Squat on the balance pad with the barbell raising	0.674	0.0219（P＜0.05）
Side throw（left）	y=0.630（±2.357）+0.579（±0.246）*Side throw (right)+0.297（±0.409）*Back throw+0.039（±0.121）*Quick v-up-0.021（±0.048）*Squat on the balance pad with the barbell raising	0.724	0.0143（P＜0.05）
Back throw	y=2.801（±1.600）+0.187（±0.257）*Side throw（left）+0.063（±0.247）*Side throw (right)+0.096（±0.091）*Quick v-up-0.048（±0.035）*Squat on the balance pad with the barbell raising	0.579	0.0465（P＜0.05）
Squat on the balance pad with the barbell raising	y=39.486 (±9.147)-0.995（±2.263）*Side throw（left）-1.410（±2.092）*Side throw (right)-3.577（±2.626）*Back throw+0.161(±0.833)*Quick v-up	0.845	0.0135（P＜0.05）
Quick v-up	y=4.936（±6.187）+0.290（±0.908）*Side throw（left）-0.215（±0.854）*Side throw (right)+1.152（±1.086）*Back throw+0.026（±0.113）*Squat on the balance pad with the barbell raising	0.627	0.2483（P＞0.05）
