# Supplementary material for: Construction of a physical fitness evaluation index system and model for high-level freestyle skiing aerials athletes in China
Source: PLoS One. 2023 Dec 8;18(12):e0295622. doi: 10.1371/journal.pone.0295622 (PMC10707543; doi:10.1371/journal.pone.0295622)
Supplement: S7 Appendix — (PDF) [file pone.0295622.s007.pdf]

S7 Appendix

Scoring results of athletes' physical fitness in the retest based on the evaluation system established in this study

Table 1 Aerials athletes' primary physical fitness indexes and comprehensive index scoring results from the first test (weighted)

	Male（n=8）				Female(n=8)				
Name	Body form	Physiological function	Physical quality	Comprehensive index	Name	Body form	Physiological function	Physical quality	Comprehensive index
C**	0.83	1.32	2.01	4.16	C**	0.83	1.06	1.53	3.42
L**	0.75	1.31	1.39	3.45	K**	0.74	1.30	1.32	3.37
L**	0.75	0.99	1.12	2.86	L**	0.87	0.96	1.22	3.05
W**	0.89	1.11	1.39	3.38	L**	0.73	0.64	1.12	2.49
Y**	0.83	1.21	1.39	3.42	S**	0.82	1.09	1.44	3.35
Z**	0.69	1.20	1.18	3.07	S**	0.87	0.73	1.04	2.64
L**	0.69	1.22	1.26	3.16	W**	0.59	0.96	1.02	2.57
Y**	0.81	1.08	1.38	3.27	Y**	1.06	0.73	1.04	2.83

Table2 Aerials athletes' primary physical fitness indexes and comprehensive index scoring results from the second test (weighted)

	Male（n=8）				Female(n=8)				
Name	Body form	Physiological function	Physical quality	Comprehensive index	Name	Body form	Physiological function	Physical quality	Comprehensive index
C**	0.83	1.44	2.01	4.27	C**	0.83	0.96	1.54	3.33
L**	0.75	1.32	1.39	3.46	K**	0.74	1.30	1.32	3.37
L**	0.75	0.99	1.24	2.98	L**	0.87	1.05	1.22	3.15
W**	0.89	1.11	1.51	3.50	L**	0.73	0.64	1.12	2.49
Y**	0.83	1.21	1.39	3.42	S**	0.82	1.09	1.35	3.27
Z**	0.69	1.22	1.18	3.08	S**	0.87	0.84	1.04	2.75
L**	0.69	1.22	1.26	3.16	W**	0.59	0.96	1.14	2.68
Y**	0.81	1.08	1.51	3.40	Y**	1.06	0.73	1.04	2.83
